# Enhanced volumetric additive manufacturing via Reversible Addition-Fragmentation Chain Transfer (RAFT) polymerization

**DOI:** 10.1038/s41467-026-73456-8

**Published:** 2026-05-27

**Authors:** Eduards Krumins, Yaxuan Sun, Long Jiang, Vincenzo Taresco, Daniel J. Keddie, Ricky D. Wildman, Hayden Taylor, Derek J. Irvine

**Affiliations:** 1 Centre of Additive Manufacturing, Faculty of Engineering, Engineering, University Park, University of Nottingham, Nottingham, NG7 2RD UK; 2 Dept. of Mechanical Engineering, University of California, Berkeley, CA USA; 3 School of Pharmacy, University of Nottingham, Nottingham, UK; 4 School of Chemistry, University Park, University of Nottingham, Nottingham, UK; 5 Department of Chemical and Environmental Engineering, Faculty of Engineering, University Park, University of Nottingham, Nottingham, NG7 2RD UK

**Keywords:** Mechanical properties, Polymers, Polymers

## Abstract

Computed Axial Lithography (CAL), a Volumetric Additive Manufacturing (VAM) technology, enables the rapid, full body i.e. not layer-by-layer, fabrication of freeform geometries within seconds through the superposition of projected light patterns. However, as conventional CAL relies on free radical polymerization (FRP), it is an intrinsically exothermic process (ΔT > 60 °C) that can trigger auto-acceleration, so compromising print fidelity and limiting scalability. By regulating polymer chain length during propagation through reversible chain transfer, Reversible Addition–Fragmentation Chain Transfer (RAFT) maintains steady, controlled reaction kinetics and prevents the sharp viscosity increase characteristic of FRP. In this study, we introduce RAFT polymerization into various (meth)acrylate-based systems within CAL to effectively mitigate heat generation and suppress auto-acceleration during photopolymerization. The success of this approach is confirmed by in-situ thermal monitoring and the suppression of thermally induced buoyancy, revealing a substantial reduction in temperature rise compared to FRP. Furthermore, RAFT chemistry enables post-printing functionalization of the printed objects, expanding CAL’s chemical versatility. This study demonstrates that RAFT-mediated CAL allows the fabrication of structures inaccessible via FRP, advancing thermally stable and functionally tunable volumetric additive manufacturing.

## Abstract (web summary)

Computed Axial Lithography is a volumetric 3D-printing method which typically uses exothermic free radical polymerisation, leading to auto-acceleration, thermal gradients and non-uniform printed objects. Here the authors used RAFT polymerisation to overcome this problem, achieving uniform samples with high resolutions.

## Introduction

Computed Axial Lithography (CAL) represents a transformative advance in volumetric additive manufacturing (VAM), offering a fundamentally layerless approach to 3D printing^1–3^. By employing tomographic reconstruction, CAL cures photopolymer resins throughout an entire 3D geometry simultaneously by projecting precisely modulated light patterns into a rotating container. This unique methodology enables both unprecedented printing speeds, i.e. on the order of ~1 min for centimetre-scale objects^4^, and removes the need for support structures, thereby eliminating post-processing steps to remove supports, minimising material waste, and facilitating the fabrication of highly complex geometries^5,6^. Furthermore, the layerless process produces parts with exceptional surface finish, mitigating mechanical weaknesses associated with inter-layer delamination^7,8^. Collectively, these attributes position CAL as one of the most promising innovations in vat photopolymerisation (VPP)-based additive manufacturing (AM). Yet, despite these advantages, scaling-up of the CAL technology remains limited, primarily due to barriers in process control which are the challenges that this work seeks to address.

**Fig. 1:**
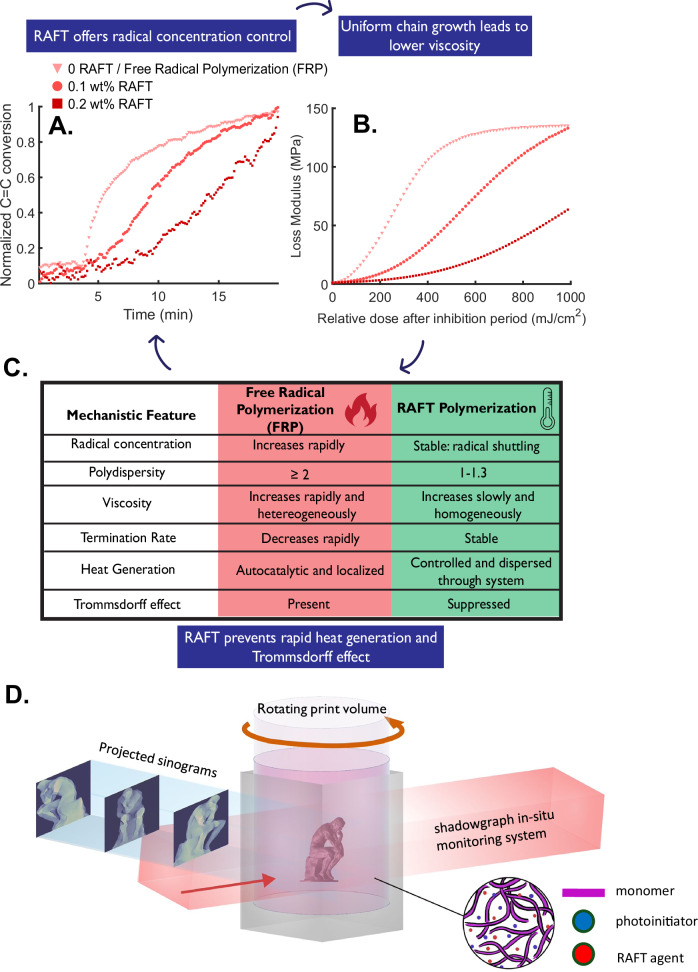
Comparison of the reaction kinetics of FRP and RAFT polymerization and their effect on the temperature and reaction kinetics of reactions using representative PETRA formulations used in CAL (see “Methods” for details). **A** UV-FTIR monitoring of the C = C bond conversion in FRP and RAFT systems, confirming successful suppression of auto-acceleration and linearisation of reaction kinetics through incorporation of RAFT. **B** UV-rheology measurements showing the evolution of the loss modulus (G″) during FRP and RAFT reactions, demonstrating suppression of rapid viscosity increase through incorporation of RAFT. **C** Scheme of mechanistic features of FRP and RAFT which contribute to their reaction kinetics. **D** Conceptual diagram showing the incorporation of RAFT-enabled resin formulations into CAL printing.

At the core of CAL lies photo-activated radical polymerization, which enables rapid volumetric solidification of complex geometries. However, the strongly exothermic nature of these reactions frequently produces the Trommsdorff, or gel, effect, leading to loss of printing fidelity^9–11^. In conventional radical polymerization (RP) (also known as free radical polymerization (FRP)), particularly when using multifunctional crosslinking monomers, polymer chain propagation leads to rapid increases in molar mass (molecular size) thereby increasing resin viscosity, whilst suppressing the rate of radical termination and leaving propagation largely unaffected^10,12,13^ (Fig. 1A, B). The resulting accumulation of active radicals accelerates polymerization further, releasing additional heat upon monomer consumption feeding back into the same cycle^9,11,14^ (Fig. 1A, B, and C). This positive feedback loop generates steep thermal gradients, which can produce thermal buoyancy-driven convection in the resin: hotter, less dense regions rise while cooler, denser regions sink, leading to resin displacement and part distortion^15–17^. At larger build volumes, limited heat and mass transfer exacerbate these instabilities, posing scalability, reproducibility, and safety challenges, including the risk of runaway reactions and ignition in flammable resin systems^9,14,18^.

Compounding these effects, the heat released during polymerization establishes temperature gradients throughout the resin, which induce spatially heterogeneous polymerization kinetics^16,19,20^. As the rates of propagation and termination steps are temperature-dependent (as per the Arrhenius relationship), warmer regions polymerise faster and generate more heat, whereas cooler regions progress more slowly^12,16,19^. Elevated temperatures also increase molecular mobility and reduce local viscosity, briefly facilitating faster chain growth^16,19,20^ (Fig. 1A, B). This leads to a continuously shifting thermal–kinetic balance: warmer regions polymerise faster and release more heat, whereas cooler zones progress more slowly. These effects produce heterogeneous crosslinking densities, internal stress gradients, and non-uniform optical and mechanical properties within a single print^19–21^.

In addition, many photo-initiators exhibit temperature-sensitive decomposition behaviour, further amplifying local reactivity^22^. Heat generated during polymerization can accelerate photo-initiator decomposition, increasing radical flux beyond that produced by light alone^23^. For example, Irgacure 819 (a Type I photo-initiator exhibits an onset of thermal decomposition near 168 °C, showing that thermal energy alone can trigger radical generation^22^. Similarly LAP (lithium phenyl-2,4,6-trimethylbenzoylphosphinate) is predominantly photo-activated, however elevated temperatures enhance its photodecomposition rate, shortening excited-state lifetimes and increasing radical formation efficiency^24^. Such thermally accelerated initiation can exacerbate temperature-driven rate disparities, particularly in thick or dense resin volumes^23^.

The exotherm associated with FRP in CAL therefore manifests as:

- Overcuring and dimensional deviation.
- Trommsdorff (auto-acceleration) behaviour.
- Buoyancy-induced convective flow.
- Reaction rate and conversion gradients.

Managing thermal excursions can be approached through both physical and chemical strategies. External cooling provides a straightforward, macroscopic means of limiting bulk temperature rise: cooling jackets, heat sinks, or conductive substrates can moderate thermal excursions^25^. However, in CAL, where stirring is prohibited to preserve optical clarity and patterned light dosage, heat dissipation occurs primarily through conduction^26^. Low thermal conductivity of typical (meth)acrylate resins limits the effectiveness of physical cooling, leaving localised hot spots and temperature-dependent kinetic heterogeneity^27^.

Heat mitigation through reversible-deactivation radical polymerization (RDRP) regulates heat generation at the molecular level. In RDRP, control agents transfer radicals in a reversible equilibrium, limiting instantaneous propagation^28–30^. Among RDRP strategies, reversible addition–fragmentation chain-transfer (RAFT) polymerization is particularly effective. RAFT has previously been explored in vat photopolymerisation and additive manufacturing contexts to enable dynamic polymer networks^31^, post-functionalisation^32,33^, and enhanced structural fidelity as well as mechanical uniformity of printed architectures^34–36^; however, these studies have primarily focused on final material properties rather than regulation of reaction kinetics or heat generation during printing. In RAFT, thiocarbonylthio chain-transfer agents mediate radical availability by shuttling between growing chains^28–30^. This reduces fast growth of polymer chain lengths during propagation via chain transfer, thereby reducing the rapid increase in viscosity which creates more uniform spatial heat profiles^28–30^ (Fig. 1). Consequently, RAFT attenuates local exotherms, improves polymerization uniformity, and enables precise control over molecular weight, dispersity, and end-group functionality for post-functionalisation and multi-material integration^28–30^.

Although RDRP methods are often considered too slow for large-scale manufacturing, if the correct RAFT agent is utilized the speed of the reaction need not be slower. Therefore, it is applicable to CAL’s rapid volumetric printing rate. Integrating RAFT into CAL provides intrinsic thermal regulation without perturbing optical or mechanical equilibrium. By suppressing exothermic spikes and associated buoyancy effects, RAFT-CAL minimizes temperature and reaction-rate gradients, ensuring uniform polymerization kinetics and high-fidelity feature reproduction across parts of different sizes and geometries within a single resin vial. This chemical control preserves CAL’s volumetric print rates while enhancing dimensional accuracy and material homogeneity, ensuring that larger features do not overcure while small, delicate features complete polymerization. Furthermore, RAFT end-group retention enables post-polymerization modification and multi-material fabrication, allowing grafting of otherwise non-photopolymerizable and spatial tuning of chemistry and mechanical properties for complex assemblies. Collectively, these mechanisms establish a strategy for thermally regulated, high-definition volumetric additive manufacturing, in which molecular-level kinetic control governs macroscopic process stability and allows complex assembly of features across scale. By coupling radical dynamics with volumetric photopolymerisation (Fig. 1D), RAFT-CAL extends CAL’s design space, enabling high-fidelity, multi-material architectures with compositional complexity and scalability previously inaccessible to FRP-based volumetric printing.

## Results

In this study, the benefits of applying RAFT in CAL were assessed with a range of commonly used AM monomers: pentaerythritol tetra acrylate (PETRA), Poly Glycerol-4 and −6 acrylates (i.e. PG4-A and PG6-A, respectively), and a 80:20 ratio mixture of a polyethylene glycol diacrylate (PEGDA) with a M_n_ 700 g.mol^−1^ and water (PEG_700_DA:H_2_O), i.e. a hydrogel formulation. These were chosen because: (a) PETRA is widely used in CAL and other photo-based AM techniques, thus is a good reference species to benchmark any RAFT induced process changes^37–39^, (b) PG4-A and PG6-A are more sustainable alternatives to petrochemically derived resins like PETRA^40^, and (c) PEG_700_DA:H_2_O because hydrogels are often printed via CAL for biomedical applications. Additionally, PG4-A and PG6-A resins exhibit high viscosities (~8000 cP), whereas the PEG700DA:H₂O mixture has a much lower viscosity (~100 cP), allowing for testing across a broad viscosity range.

One of the key features of RAFT polymerization is its ability to control the polymerization of a wide range of functional monomers^28,41^. However, the monomer compatibility of RAFT is strongly influenced by the reactivity of the chain-transfer agent (CTA), and careful selection of the CTA molecular structure is required to achieve optimal control in conventional solution-based RAFT polymerization^28,41^. As a result, a set of established general rules exists to guide the selection of appropriate RAFT agents for specific monomers or monomer combinations. A brief discussion of these solution-based RAFT guidelines is provided in the Supplementary Information (S1). In this paper, five RAFT agents with a range of reactivity towards propagating radicals (i.e., transfer coefficients (C_tr_)) were screened for their efficacy in controlling the polymerization of these chosen acrylate-based (macro)monomers. These were, in order of decreasing C_tr_, a dithiobenzoate (2-cyano-2-propyl dibenzothioate (**1**, CPBD)), three trithiocarbonates with radical re-initiating R-groups of various stability (4-cyano-4-[(dodecylsulfanylthiocarbonyl)sulfanyl]pentanol (**2**)) 2-cyano-2-propyl dodecyl trithioate (**3**), 2-dodecylthiocarbonylthioylthio-2-methylpropionic acid (**4**), and a dithiocarbamate (cyanomethyl methyl(phenyl)carbamodithioate (**5**)) (Fig. S1 (A)). The screening involved conducting model photopolymerizations in glass vials, to mimic the polymerization conditions of CAL printing, and following the temperature evolution during both photo-polymerization and UV-Differential Scanning Calorimetry (UV-DSC). The screening revealed that chain transfer with the trithiocabonates **2**-**4** and dithiocarbamate **5** was not rapid enough to mitigate the temperature evolution through autoacceleration during photopolymerization; the temperature increased by ~20 °C during the reactions (Fig. S1 (B)). Meanwhile, the most active dithiobenzoate RAFT agent CPBD **1** dissipated the temperature evolution during the reaction, so the temperature increased by only ~10 °C in bulk polymerization (Fig. S1 (B) and S2). It is clear that the very high C_tr_ of CPBD allows it to keep poly(acrylate) propagating species shorter for an extended time, effectively mitigating the autoacceleration exotherm during photopolymerization of the acrylate-based monomers, PG6-A and PETRA. As such, CPBD was used in all further RAFT-mediated photopolymerizations.

To further validate the efficacy of CPBD similar tests were performed with PEGDA. The photopolymerization data showed that loadings of 0.05–0.2 wt% CPBD, when 0.1 wt% of LAP photo-initiator was used, mitigated the PEGDA exotherm (Fig. S3). Meanwhile, the PEGDA UV-DSC data showed heat generation could be mitigated using CPBD, where the level of CPDB required increased as the LAP loading increased (Fig. S4–S6). Dodecanethiol, a non-RAFT non-reversible thiol control agent, was also tested and found to be ineffective for CAL as even with relatively large amounts (~2.5 wt%) it showed no control over the exotherm (Fig. S7–S8). Hence, the reversible nature of RAFT was demonstrated to be key to delivering control to CAL.

**Fig. 2:**
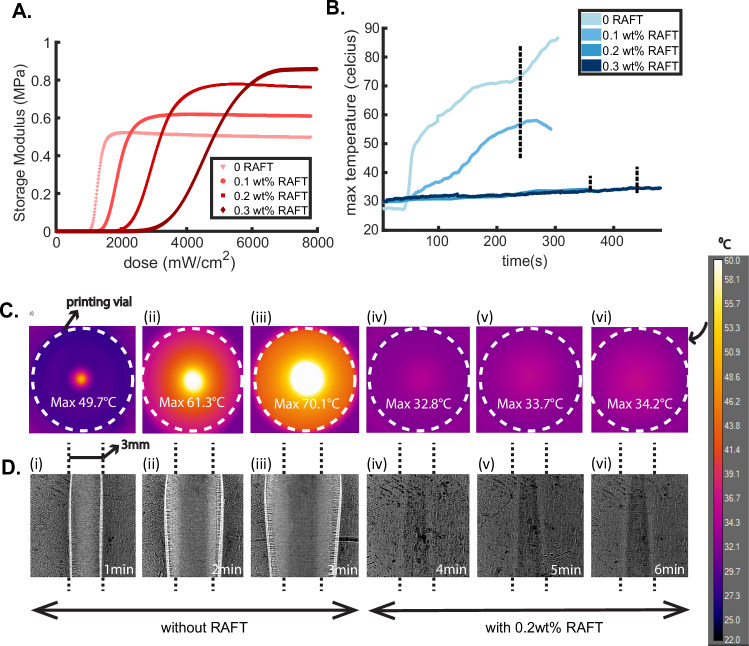
Effect of RAFT polymerization on the reaction rate of CAL and temperature evolution. **A** Photo-rheology of PETRA-based formulations from 0 wt% to 0.3 wt% of CPDB **1** RAFT agent. The depicted storage modulus is averaged data over *n* = 3 samples. **B** Temperature changes during CAL printing of PETRA-based formulations from 0 wt% to 0.3 wt% of CPDB **1** RAFT agent. The dotted black lines denote the extent of light projection. **C** IR thermal imaging for PETRA (i–iii) without RAFT and (iv–vi) with 0.2 wt% CPDB **1** RAFT agent. **D** Shadowgraph for PETRA (i–iii) without RAFT and (iv–vi) with 0.2 wt% CPDB **1** RAFT agent when printing a 3 mm cylinder. (i to iii) In C and D show three time points of free radical polymerization—(1 min) the onset of print formation, (2 mins) the start of overcuring, (3 min) significant overcuring. (iv–vi) Show three time points of RAFT controlled polymerization—(4 min) the onset of print formation, (5 min) finish printing of predetermined structure, (6 min) no overcure 1 min after the print shape is defined. Note the accuracy and thermal sensitive of the IR camera used is ±5% and a thermal sensitivity/NETD of <0.05 °C.

Photo-rheology conducted on the PETRA-based samples comparing the time taken for the storage modulus to plateau revealed that whilst RAFT successfully reduced reaction rates compared to non-RAFT samples, all the rates were rapid enough to work in the time frame commonly used in CAL (Fig. 2A). Direct measurement of the degree of conversion was conducted using UV-FTIR, which confirmed trends observed in the photo-rheology data for both PETRA and PEGDA-based samples (Fig. S11). Additionally, photo-rheology revealed that the RAFT mediated printed objects had enhanced mechanical properties (Fig. 2A and S10), possibly due to the RAFT process leading to more uniform distances between cross-links and/or greater homogeneity throughout the samples^42^. Incorporation of RAFT agents retards the polymerization process, reducing kinetic trapping during network formation. This leads to a more efficiently packed polymer network with a lower and more homogeneous free-volume distribution which can be linked to enhanced mechanical performance in thermoset polymers^43^. Furthermore, as RAFT agent concentration increases, the influence of kinetic control on free-volume reduction is expected to diminish once a limiting packing state is reached, resulting in a plateau in mechanical properties observed in the data^44,45^.

IR-thermal imaging was performed on the PETRA-based formulations using CPBD as the RAFT agent, to enable measurement of the heat evolved during printing of a specific design, which consisted of a cylinder with a 3 mm diameter and a height of 20 mm. This design was chosen because the heat from the top of the part could be recorded via IR thermal imaging using the apparatus shown in Fig. S12. A series of measurements were made to determine the influence on the reaction temperature of systematically increasing the RAFT agent concentration. A clear trend was exhibited in these experiments. Samples prepared in the absence of RAFT agent (i.e., 0 wt% CPBD **1**) exhibited a large temperature increase of +59 °C during photopolymerization (Fig. 2B). Upon addition of 0.1 wt% CPBD **1** the temperature evolution from an analogous reaction was reduced to +27 °C. Moreover, with a 0.3 wt% concentration limiting the temperature increase during reaction to just +3.5 °C. (Fig. 2B).

Furthermore, to support the proposition that RAFT-mediated VAM can successfully mitigate heat accumulation in scale-up, a physics-based and conduction-limited thermal scaling analysis was performed using calibrated data from the in-situ infrared temperature measurements made during our experimental program for representative PETRA formulations. This model predicted that once the object size exceeds the characteristic thermal diffusion length, governed by the printing time and the thermal diffusivity, the peak temperature rise becomes dominated by the local volumetric heat-generation rate and was only weakly dependent on the overall object scale. See detailed discussion of simulations conducted in discussion S7 which includes Figs. S13 and S14.

To further investigate the effect of RAFT on auto-acceleration events during photopolymerization, thermal imaging was continued after print completion to assess if polymerization of the different samples would continue without external stimuli. For the 0 and 0.1 wt% CPBD **1** RAFT samples the projections were stopped after 240 s. The 0 wt% RAFT sample continued generating heat as seen by the increase of temperature between 240 and 300 s (Fig. 2B), indicating this sample underwent ‘dark cure’, further evidence that the FRP reaction had reached a point of auto-acceleration. The sample with 0.1 wt% CPBD **1** RAFT exhibited a small increase in temperature from 240 to 265 s, after which a sharp drop in temperature was seen (Fig. 2B). The samples with 0.2 wt% and 0.3 wt% had slower reaction rates, hence the printing time was extended to 400 s. However, only a small increase in temperature was seen after this period (Fig. 2B). These observations clearly illustrate RAFT polymerization mitigates auto-acceleration in CAL printing.

Shadowgraph monitoring revealed that the sample containing 0.2 wt% RAFT exhibited no overcuring even 2 min after the onset of object appearance, whilst the 0 wt% RAFT sample overcured quickly after reaching its predetermined design (Fig. 2C, D). This again demonstrated that relatively small amounts of RAFT agent could greatly reduce the exotherm caused by the photocuring of (meth)acrylates used in CAL. Our data suggests that the manufacturing method does not fundamentally alter the underlying characteristics of the RAFT mechanism. Accordingly, general guidelines for RAFT-agent selection based on monomer chemistry are expected to remain applicable in CAL printing.

**Fig. 3:**
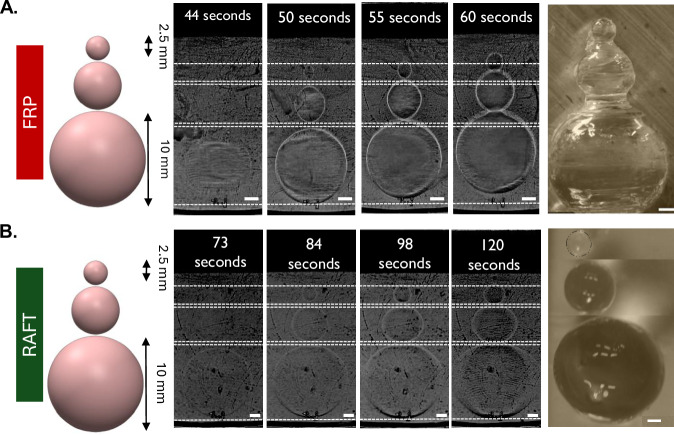
Mitigation of the thermal buoyancy issue in VAM via the use of RAFT polymerisation. **A** Simultaneous fabrication of objects with different feature sizes via FRP. Left: illustration of the printed part: three spheres with dimensions of 10 mm, 5 mm, and 2.5 mm with three-pixel gaps between the spheres in the z-direction. Center: images taken from in-situ monitoring of the FRP-VAM print, showing formation of three spheres and subsequent rising of the parts due to thermal buoyancy. Right: the result of CAL printing via conventional radical polymerization, showing spheres fused together. **B** Simultaneous fabrication of objects with different feature sizes via RAFT. Left: illustration of the printed part: three spheres with dimensions of 10 mm, 5 mm, and 2.5 mm with three-pixel gaps between the spheres in the z-direction. Center: images taken from in-situ monitoring of the RAFT-VAM print, showing formation of three spheres with no rise of parts showcasing successful mitigation of thermal buoyancy during VAM printing. Right: the result of CAL printing via conventional radical polymerization, showing spheres fused together. Scale bars, 1 mm.

To evaluate the ability of RAFT polymerization to mitigate buoyancy-driven instabilities, a model design consisting of three spheres of different diameters (10 mm, 5 mm, and 2.5 mm) separated by 150 µm ( ≈ 3 pixels) in the z-direction was used (Fig. 3). When printed using a conventional FRP PETRA-based resin, this structure could not be fabricated as intended: thermal expansion of the curing 10 mm sphere caused it to rise prematurely, displacing the smaller spheres and resulting in a single fused structure (Fig. 3A, middle; Supplementary Movie 1). In contrast, the PETRA resin containing 0.1 wt% RAFT agent reduced the exothermic response during polymerization, suppressing buoyancy-driven motion. Consequently, all three spheres formed within a more uniform time window and retained their designed 150 µm spacing (1.5% of the largest sphere’s diameter) without fusion (Fig. 3B; 2), demonstrating precise control and improved print fidelity. The RAFT-printed spheres also exhibited smoother, striation-free surfaces, consistent with slower, more uniform polymer growth and a more homogeneous network (Fig. 3)^46^. A faint peripheral halo occasionally observed in RAFT prints likely originates from a low-conversion region near the surface, which can be removed during post-processing to yield parts closely matching the CAD design (Fig. 3B). An aim of this work was to increase volumetric packing density and enable multipart printing. Thus, the relevant measure of resolution was defined as the minimum distance that could be achieved between parts printed simultaneously that remained independently resolved. For the apparatus used in this study, this was determined to be 150 µm using the RAFT system, corresponding to three projected pixels (voxels), as seen in Fig. 3.

**Fig. 4:**
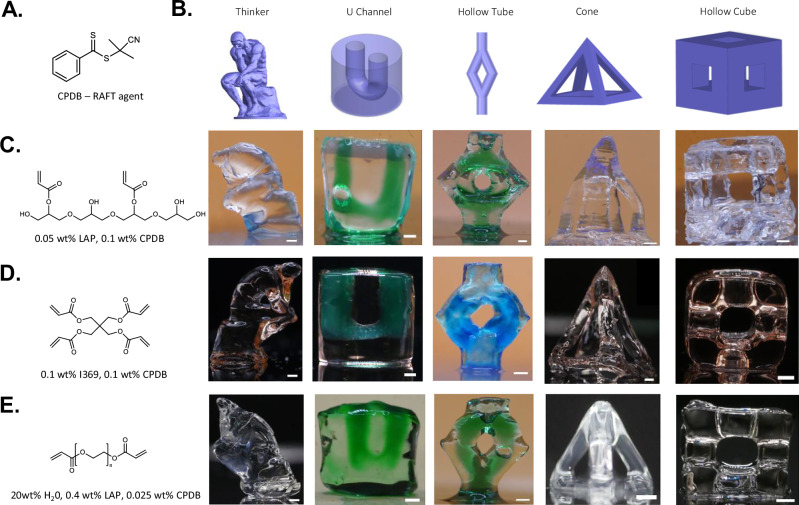
The results of RAFT-based CAL printing for a variety of designs and polymer systems. **A** Structure of RAFT agent used: CPBD. **B** CAD models for the five designs (Thinker, U-Channel, Y-tube, Pyramid trusses, and Hollow cube) used for CAL printing fidelity testing. Green or blue dyed deionised water has been used in the U-channel and Y-tube structures to show interior hollow passages. **C** Structure of Polyglycerol 4 acrylate and the optimal RAFT agent and photo-initiator concentrations. Images of the five printed structures composed of PG-4A with 0.1 wt% CPBD and 0.05 wt% LAP. **D** Structure of PETRA and the optimal RAFT agent and photo-initiator concentrations. Images of the five printed structures composed of PETRA with 0.1 wt% CPBD and 0.1 wt% I369. **E** Structure of PEGDA 700 with water (80/20 mixture respectively) and the optimal RAFT agent and photo-initiator concentrations. Images of the five printed structures composed of PETRA with 0.025 wt% CPBD and 0.4 wt% LAP. Scale bars, 1 mm.

To test the suitability and applicability of RAFT polymerization in CAL across a range of materials, three materials systems were chosen as tests. Each of these materials were used to print five different structures. These structures were chosen because they would test the ability of RAFT polymerization to print objects with small positive and negative features as well as objects with overhanging angles and other challenging geometric features. The printing parameters and concentration of the RAFT agent were optimised for each material system (Fig. S15 and Tables S1–S4). The optimal RAFT agent concentration varied in line with the changing effectiveness of the RAFT agent (CPBD) with different materials. Optimal printing conditions and concentrations of initiators allowed for the successful printing of all five structures for each of the material systems (Fig. 4). This shows that RAFT polymerization can be used to effectively print a variety of resins with vastly different viscosities, molecular structures, and physical properties via CAL.

**Fig. 5:**
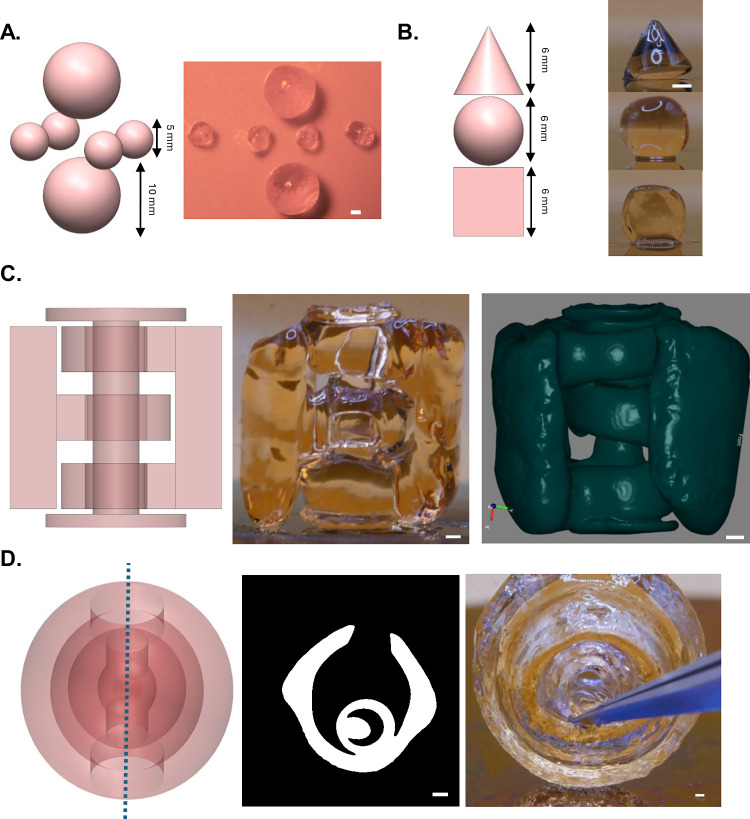
Expansion of design freedom in CAL via RAFT demonstrated using PETRA 0.1 wt% RAFT agent. **A** Increase of volume packing of objects in CAL via printing objects with various feature sizes centered on and away from the rotational axis. Two 10 mm spheres at the top and bottom along the central rotational axis and four 5 mm spheres on separate axes in the middle of the z-axis. The *z*-axis separation is three pixels. **B** Construction of different geometries (a cone, a cube, a sphere) with three-pixel gaps in the *z* direction. **C** Construction of a hinge-type interlocked structure (Supplementary Movie 7). From left to right: CAD illustration of the printed part, and micro-CT-scanned reconstruction. **D** Production of a three-layered nested ovoid/spherical structure. From left to right: CAD illustration of the printed part, a slice of the middle segment of the part taken via micro-CT, and printed part. Scale bars: 1 mm.

This work also aimed to increase the design freedom of CAL through RAFT polymerization. When printing an object located along the central axis of rotation of the resin vat and positioned at the optimal focal distance of the light source, features as small as ~20–80 µm, both positive and negative, can be successfully reproduced with standard FRP based resins^7^. However, when an object with distinctly different feature sizes is printed it is difficult to obtain adequate resolution for both the small and large features as they have vastly different curing times. This problem is accentuated if objects are printed simultaneously with different central axes. Thus, designs were printed with the optimised PETRA-based RAFT formulation to evaluate the ability to enhance the technique’s freedom of design (Fig. 5).

A design composed of six separate spheres was chosen to investigate the potential of CAL to produce separate parts at high packing density (Fig. 5A). The design had two 10 mm spheres which were printed along the central axis of the vial. Additionally, in the vertical gap between the 10 mm spheres, four 5 mm spheres separated by 6 mm in the *x* and *y* axis were printed offset from the central axis of the vial (Fig. 5A). The vertical spacing between the 10 mm spheres and the middle 5 mm spheres is 150 µm (Fig. 5A). The ability to print differently sized parts in various radial positions within the printing volume greatly increases the volume packing potential of CAL (Fig. 5A). The printing of these spheres was successful, as both the 10 mm and 5 mm spheres formed simultaneously and did not exhibit overcuring during printing (Supplementary Movie 3). Although some halos are visible in the shadow-graph Supplementary Movies 1–6 between printed regions of the RAFT-printed objects, they correspond to low-conversion polymers, as the fully cured regions exhibit a greater refractive index change (darker hue in the Supplementary Movies). These outer halos are easily removed during post-printing washing. After post-processing, the dimensions of the parts were found to be consistent with the CAD design (Fig. 5B). Its FRP counterpart, printed with the same set of projections, can be found in Figure. S16 and Supplementary Movie 4, where the poor printing of the final product can be observed directly.

To demonstrate the versatility and design freedom of the technique, three different shapes: a cone, a cube, and a sphere were printed simultaneously (Fig. 5B). The three shapes were designed to be of the same height (6 mm) and they were separated by 150 µm in the *z*-direction. The three shapes printed successfully and exhibited no overcuring (Supplementary Movie 6), whereas their FRP counterparts fused all together once again (Fig. 5B, Fig. S17, Supplementary Movie 5). Together these results show that RAFT can be used to print objects of different sizes and shapes at various radial positions within the printing volume (Fig. 5A, B).

A hinge-type structure was used to investigate the possibility of printing interlocking parts via RAFT-mediated CAL. This capability is of importance because similar design requirements are needed to produce key industrial components such as interlocking gears and a variety of bearings. The hinge structure had a pin in the middle of the part with two stoppers at either end. One side of the hinge has a hollow cylinder wrapped around the middle of the pin, whilst the other side of the pin is connected to the top and bottom of the pin, with both stoppers keeping it in place (Fig. 5C, left). The part was printed successfully and did not overcure (Fig. 5C, middle). Both sides of the hinge could freely rotate around the central pin (Supplementary Movie 7), showing that RAFT-mediated CAL can be used to print interlocking parts whilst retaining freedom of movement. A micro-CT scan of the hinge structure is shown in Fig. 5C (right). Lastly, a “nesting doll”-type design was created to test whether RAFT-mediated CAL could be used to manufacture fully formed structures within other structures during the same print (Fig. 5D). The chosen design was composed of three ovoid/spherical items which were nested. The largest ovoid shell had a diameter of 18 mm and a wall thickness of 2.5 mm. The second hollow ovoid shell was designed to be in the middle of the empty space within the larger shell. A 2.5 mm gap was left between the two ovoids and the second ovoid had a 10 mm diameter and a wall thickness of 2.5 mm. The innermost object was a solid sphere with 2.5 mm diameter, located inside the hollow space of the second ovoid; a 2.5 mm gap was left between the second and third structures (Fig. 5D, left). The use of RAFT polymerization prevented the ovoids/spheres from overcuring before all the structures were formed. The post-printing washing step revealed a “nesting doll”-type spherical structure composed of two ovoids with one smaller sphere in the middle of the interior (Fig. 5D, right). A cross-sectional slice of its micro-CT scan can be seen in Fig. 5D (middle).

**Fig. 6:**
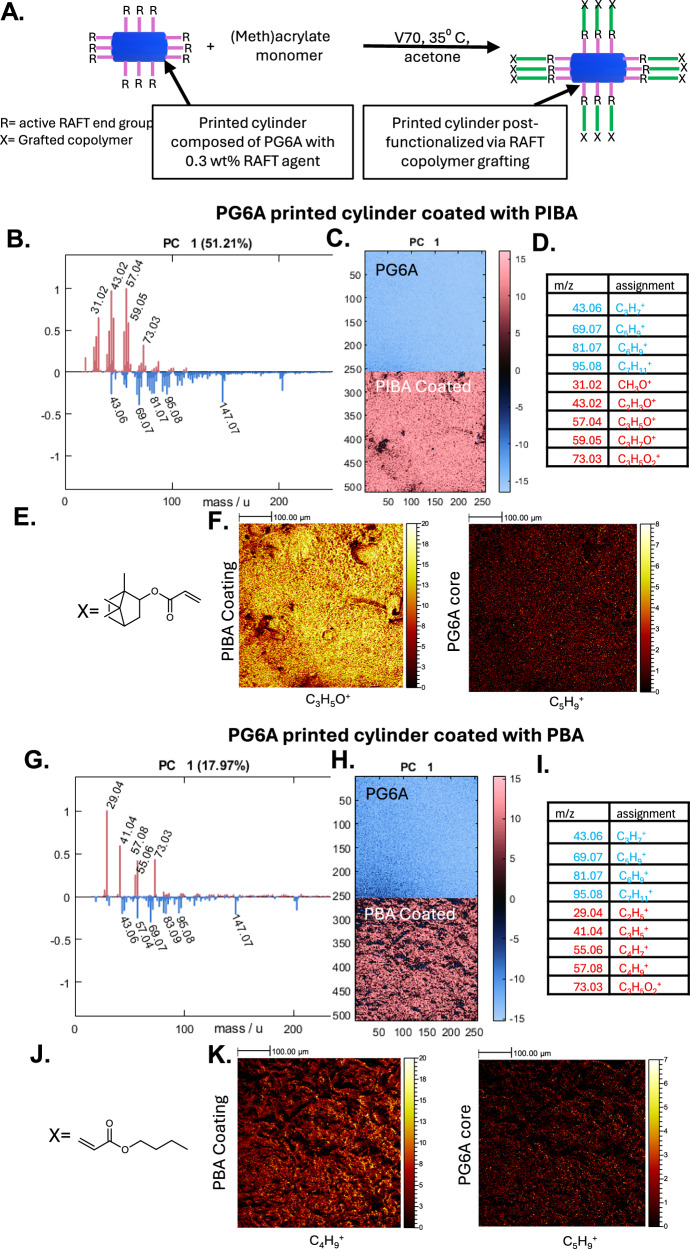
Post-Printing Functionalization of CAL-printed parts composed of PG6A with 0.3 wt% RAFT agent (CPBD). **A** Reaction scheme for post-printing functionalisation through block co-polymer synthesis. **B–F** ToF-SIMS results of a PG6A printed part with PIBA grafted at the surface via RAFT polymerization: **B** spectral results, **C** image results, and **D** list of representative ions for PIBA (red) and PG6A (blue) of the first principal component (PC1) results of the ToF-SIMS positive ion data of the uncoated and PIBA coated PG6A. **E** Structure of IBA. **F** ToF-SIMS mapping of C_3_H_5_O^+^ which is a representative of PIBA and of C_5_H_9_^+^ which is a representative of PG6A. G-K.) ToF-SIMS results of a PG6A printed part with PBA grafted at the surface via RAFT polymerization: **G** spectral results, **H** image results, and **I** list of representative ions for PBA (red) and PG6A (blue) of the PC1 results of the ToF-SIMS positive ion data of the uncoated and PBA coated PG6A. **J** Structure of BA. **K** ToF-SIMS mapping of C_4_H_9_^+^ which is a representative of PBA and of C_5_H_9_^+^ which is a representative of PG6A.

The use of RAFT polymerization can increase the applicability and versatility of CAL through post-printing functionalisation. One of the benefits of RAFT polymerization is that it leaves a “living” end group on the polymer chain. These end groups have been used to synthesise block co-polymers and/or add functional (e.g., biologically active) end groups^47–51^. Two different monomers, butyl acrylate (BA) and isobornyl acrylate (IBA), were chosen to react with the “living” end group on the surface to grow polymeric chains from the surface of the printed parts (Fig. 6). This would show that a broad range of functionalities can be added to the printed part and achieve multi-material processing.

The block copolymers on the surface of the parts were synthesized by immersing the printed part into a compatible solvent and temperature (Fig. 6A) and conducting a thermal polymerization. Post polymerization, any possible homopolymer/impurities were removed via Soxhlet extraction. Then ToF-SIMS analysis was performed on the PG6A printed parts, which were now proposed to be coated with PBA and PIBA, to define if the multi-material structure had been successfully fabricated. Principal component analysis (PCA) was performed on the ToF-SIMS positive ion data of the uncoated and the PIBA coated samples and showed a significant chemical variance of ~50% between the uncoated and PIBA coated PG6A printed samples (Fig. 6B, C). The analysis revealed key representative ions of the PIBA coating and the PG6A printed core (Fig. 6D). The ion images show that the coating is quite even on the part surface and that the map of the representative ion of PIBA is more intense than that of the PG6A core, therefore the coating via RAFT induced block copolymerization of IBA was successful (Fig. 6F).

ToF-SIMS analysis was also performed on the PG6A printed part coated with PBA. This coating was also successful as PCA showed a ~17% variance between the PBA-coated sample and the uncoated PG6A printed part (Fig. 6G, H). ToF-SIMS analysis revealed ions associated with PBA to be on the surface of the coated printed part (Fig. 6I). These ions were evenly spread across the surface of the mapped region, showing that the block copolymerization coating occurred across the entirety of the surface (Fig. 6K). The ion map of the PBA representative is more intense than that of the PG6A core, further showing that the RAFT-mediated coating process was successful (Fig. 6K). DSC was performed on the coated and uncoated PG6A printed parts, revealing that glass transition temperatures characteristic of PBA and PIBA are present in the coated samples (Fig. S19 and S20). These two polymerisations show that it is possible to functionalise RAFT-containing parts post-printing with a variety of monomers. Additionally, the coating with PIBA shows that functional polymers can be attached via this method, as PIBA is known to be anti-biofouling. This could lead to the production of multi-functional and multi-material parts via CAL. However, we note that stable post-print functionalisation under various post-curing, storage, or application conditions will require further optimisation of processing conditions.

## Discussion

This study demonstrates that integrating RAFT-mediated controlled radical polymerization into Computed Axial Lithography (CAL) offers a powerful strategy to overcome the auto-acceleration challenges inherent to rapid radical polymerization. This strategy allows the user to suppress thermal buoyancy-based printing inaccuracies and by tuning RAFT agent concentrations across diverse polymer systems, we achieved enhanced process control that enables the fabrication of complex geometries without compromising rapid fabrication speed. Notably, simultaneous multi-part printing with separations as small as ~150 µm (~3 pixels) was successfully realised highlighting the potential for high-resolution, densely packed architectures. Polymerization kinetics, monitored via photo-rheology, UV-FTIR, and UV-DSC, confirm that the RAFT approach affords a tunable inhibition period, controllable curing rates, and adjustable mechanical properties. In-situ thermal imaging further verifies the suppression of excessive temperature rises, mitigating heat-driven instabilities and distortion. The ability to regulate polymerization exotherms and kinetics expands CAL’s design space, enabling the creation of intricate, closely packed, or nested multi-material structures that were previously inaccessible. Furthermore, the living character of the RAFT end groups unlocks versatile post-functionalisation strategies, broadening the functional landscape of CAL-printed objects. These advances pave the way for next-generation applications, including hydrogel-based drug delivery devices and precisely engineered micro-reactors. This is because the benefits of conventional CAL over layer-by-layer printing approaches, e.g. ultrafast printing speed, freedom of geometry, and mitigation of mechanical weaknesses associated with inter-layer delamination, are retained in RAFT-mediated CAL. Furthermore, the improved resin stability and the presence of surface located active RAFT headgroups could lead to the 4D-printing of actuators capable of dynamic shape transformation via post-grafting. Collectively, this work establishes RAFT-controlled CAL as a robust platform for high-definition volumetric manufacturing and advanced materials engineering.

## Methods

### Materials

Pentaerythritol tetra acrylate (PETRA), polyethylene glycol diacrylate (PEGDA), Lithium phenyl-2,4,6-trimethylbenzoylphosphinate (LAP), 2-Benzyl-2-dimethylamino-1-(4-morpholinophenyl)-butanone-1 (I369), Diphenyl(2,4,6-trimethylbenzoyl)phosphine oxide (TPO), 2-Cyano-2-propyl benzodithioate (CPBD), 2-cyano-2-propyl dodecyl trithioate, 2-dodecylthiocarbonylthioylthio-2-methylpropionic-acid, 4-cyano-4-dodecyl-sulfanyl-pentanol, Cyanomethyl methyl(phenyl) carbamodithioate, and isobornyl acrylate were purchased from Sigma Aldrich. Acetone, Methanol, and Tetrahydrofuran were purchased from Fischer Scientific. 2,2′-azobis(4-methoxy-2.4-dimethyl valeronitrile) was purchased from Wako Pure Chemical Industries, Ltd. Butyl acrylate was purchased from Kaneka. Poly Glycerol-4 and 6 acrylate (PG6-A) are synthesized in house based on a published protocol^40^.

### Formulation of resins for printing and post processing

The chosen (meth)acrylate (10 g) was added to a glass vial. To the vial the chosen photo-initiator (e.g. LAP, TPO, Irgacure 316) (0.25 wt%−1 wt%) and RAFT agent (0.025–1 wt%) were added. The solution was then sonicated for 30 min and mixed using a magnetic bar on top of a magnetic plate. After printing, the printed parts are rinsed using isopropyl alcohol (IPA) or distilled (DI) water to remove any residual resin and flood cured using a commercial post-curing chamber (Formlabs Form Cure).

### Post-functionalisation reaction

2,2′-azobis(4-methoxy-2.4-dimethyl valeronitrile) (V70) (0.5 wt%, 0.025 g, 0.08106 mmol) was added to tetrahydrofuran (THF) (5 ml), then the chosen monomer was added (5 g; Butyl acrylate: 0.039 mol, Isobornyl acrylate: 0.024 mol) and the mixture was allowed to stir for 15 min at 250 rpm. A printed part composed of Poly Glycerol 4 – acrylate with 0.3 wt% CPBD RAFT agent was immersed into the solution and the stirring rate lowered to 50 rpm. The mixture was heated to 35 °C for 24 h. The mixture was then cooled and then added dropwise to ice-cold methanol to induce precipitation, including the printed part. The printed part was then washed with THF (3×) and left to dry for 30 min. The printed part was then washed via Soxhlet extraction for 24 h with acetone at 90 °C. The resulting coated printed part was then collected and left to dry.

### Temperature monitoring during photopolymerization of (meth)acrylate monomers

Into a glass vial the chosen (meth)acrylate monomer (1 g) was added. To the vial the chosen photo-initiator (e.g., LAP, TPO, Irgacure 369) (1 wt%, 0.01 g) and RAFT agent (1 wt%, 0.01 g) were added. The solution was then sonicated for 30 min and then placed into the VAM printer. A thermocouple was placed into the middle of the vial, and the vial was then rotated at 6 rpm. A 405 nm projector then illuminated the rotating vial for 10 min, whilst temperature was recorded every 15 s.

### Differential scanning calorimetry (DSC)

Measurements were performed using a TA Instruments Q2000 Differential Scanning Calorimeter at a heating rate of 20 °C min⁻¹ over a temperature range of −70 to 200 °C (the temperature range was adjusted slightly depending on the sample). Samples (5 – 10 mg) were sealed in hermetic aluminium pans with lids (TA Instruments), with an empty pan used as reference. The DSC cell was continuously purged with nitrogen at a flow rate of 50 mL min⁻¹.

### Photo-differential scanning calorimetry (photo-DSC)

Measurements were performed using a PerkinElmer DSC 8500 coupled with an OmniCure Series 2000 lamp. Resin samples were prepared following the procedure described in the Formulation of Resins for Printing and Post-Processing section. A single drop of each formulation was pipetted into an open standard aluminium DSC pan (PerkinElmer). Samples were allowed to equilibrate (with lid closed to prevent UV ingress) in the instrument prior to illumination with the OmniCure light source to initiate photopolymerisation. Thermal data were collected and analysed using Pyris Manager.

### CAL printer setup

All CAL printing shown in the main text was performed using a custom-built printer equipped with a EVM projector unit (405 nm) from Keynote Photonics, coupled to a plano-convex lens with a focal length of 180 mm. The light intensity of 405 nm projection at focal plane is 15.5 mW/cm^2^. A rotational rate of 24°/s was used. The projected pixel (square) size at focal plane is 50 µm. The in-situ monitoring of printing process through shadow-graph was achieved with a previously published setup integrated with the CAL printer used^52^. Photoshop (Adobe, USA) was used to enhance contrast of the shadowgraphs showed in Fig. 3D using the “Levels” function. The prints shown in Fig. S15 were performed using a separate CAL setup equipped with a 405 nm projector from Wintech Digital Systems Technology, providing a pixel size of 19 µm at the focal plane. All projections are optimised using the OSMO algorithm with an upper threshold of 0.9 and a lower threshold of 0.3^53^. Any intensity scaling is applied by multiplying the pre-calculated, normalized sinogram (with a maximum intensity of 1) by a scaling factor (as shown in the tables in SI), re-normalizing the resulting sinograms to the new maximum intensity, and truncating any values exceeding 1 back to 1.

### Thermal imaging

Thermal imaging was conducted using a FLIR A65sc Test Kit. FLIR ResearchIR software was used to record real time data which was then exported and analyzed using Matlab R2023b. The accuracy and thermal sensitive of the IR camera is ±5% and <0.05 °C. The spectral range of this camera is 7.5–13 µm, and thus UV light projection and white light source used in our shadow-graph setup do not perturb the imaged temperature.

### Photorheology

Photorheology tests (see setup in Fig. S9) were performed on an Anton Paar Physica MCR301 rheometer with 25 mm diameter upper parallel plate. A custom LED accessory (center wavelength of 404 nm) was attached to the lower plate of the rheometer where the 404 nm LED was placed underneath a microscope glass slide on top of which the photopolymer sample to be tested was dispensed^54^. The light intensity measured right on top of the glass slide was 18.5 mW/cm^2^. Oscillatory shear loading was performed on each material formula in room temperature with an amplitude of 1% strain and frequency of 1 Hz. The parallel plate gap was set to 1 mm. After initiating the oscillatory measurement, a 60-s dark period was employed to measure both the storage modulus (G′) and loss modulus (G″) at zero degree of curing. These initial values served as the baseline for the subsequent time sweep, during which the LED light was activated to cure the material until it reached a plateau modulus. Three independent replicates were prepared for each tested material, and the results were averaged. For visual clarity, Figs. 1B and 2A displays every second data point from the dataset.

### UV Fourier Transform Infrared Spectroscopy (FTIR)

UV-FTIR spectroscopy was conducted using a Nicolet iS50 FTIR spectrometer with an MCT detector (Thermo Fisher Scientific, USA) to monitor the photopolymerization process in real time. To ensure synchronised curing conditions, the same LED light source employed in the photorheology experiments was utilized. Samples were prepared by sandwiching the material between two infrared-transparent salt plates, with six layers of Kapton tape used as spacers to maintain a consistent film thickness. Spectra were collected over the mid-infrared range (4000–400 cm⁻¹) at a resolution of 1 cm⁻¹, with a sampling rate of 100 Hz, over a 20-min period. A 45-s dark period preceded the measurement to establish a baseline signal. Particular attention was given to the absorption band at 1952 cm⁻¹, which corresponds to the C = C = N stretching vibration characteristic of ketenimine groups. Monitoring the intensity changes at this wavenumber provided insights into the consumption of reactive functional groups during the curing process, thereby enabling the assessment of curing kinetics and the extent of polymerization under the applied UV conditions. For visual clarity, Fig. 1A displays every 15th data point from the dataset.

### Micro-CT scan

Glidewell Restorative CT with suffix of 50 µm was implemented for CT scans. 3D Slicer is then used to reconstruct the complete object and extract cross sections.

### ToF-SIMS

The ToF-SIMS data were acquired using a ToF-SIMS V instrument (ION-TOF GmbH., Münster, Germany). Primary Ion Beam was a bismuth liquid metal ion gun (Bi3 + ) run at 25 keV (pulsed target current of ~0.03 pA). Data was acquired over regions of 500 × 500 µm at 256 × 256 pixel resolution. Data analysis was performed using SurfaceLab 7 software (IONTOF GmbH).

A principal component analysis (PCA) of the SIMS image data was performed using simsMVAsoftware^55^. (It offers the capacity to summarise key variance in an ion map and correlate surface patterns to ion data.) Before the PCA was conducted, the ion image was normalised to the total ion image to minimise the topographic effect. Mean centering was applied for the PCA analysis.

## Supplementary information

Supplementary Information

Description of Additional Supplementary File

Supplementary Movie 1

Supplementary Movie 2

Supplementary Movie 3

Supplementary Movie 4

Supplementary Movie 5

Supplementary Movie 6

Supplementary Movie 7

Transparent Peer Review file

## Data Availability

All data generated or analyzed in this study are included in the main manuscript and the Supplementary Information. No restricted or confidential datasets were used. No data requiring deposition in a public repository was produced. All data are available from the corresponding author upon request.
