# Supplementary material for: Enhanced volumetric additive manufacturing via Reversible Addition-Fragmentation Chain Transfer (RAFT) polymerization
Source: Nat Commun. 2026 May 27;17:6907. doi: 10.1038/s41467-026-73456-8 (PMC13389418; doi:10.1038/s41467-026-73456-8)
Supplement: Supplementary file 1 — Supplementary Information [file 41467_2026_73456_MOESM1_ESM.docx]

**Supplementary Information**

**Enhanced Volumetric Additive Manufacturing via Reversible Addition-Fragmentation Chain Transfer (RAFT) Polymerization**

Eduards Krumins^a,‡^, Yaxuan Sun^b,‡^, Long Jiang^c^, Vincenzo Taresco^d^, Daniel J. Keddie^d^, Ricky D. Wildman^a,e*^, Hayden Taylor^b,*^ and Derek J. Irvine^a,e.*^

^a^ Centre of Additive Manufacturing, Department of Chemical and Environmental Engineering, University Park, University of Nottingham, Nottingham, NG7 2RD.

^b^ University of California, Berkeley, CA 94720-1740

^c^ School of Pharmacy, University of Nottingham, Nottingham, NG7 2RD, United Kingdom

^d^ School of Chemistry, University Park, University of Nottingham, Nottingham, NG7 2RD.

^e^ Department of Chemical and Environmental Engineering, University Park, University of Nottingham, Nottingham, NG7 2RD.

‡ These authors contributed equally to this work

* Corresponding authors addresses: [derek.irvine@nottingham.ac.uk](mailto:derek.irvine@nottingham.ac.uk), [ricky.wildman@nottingham.ac.uk](mailto:ricky.wildman@nottingham.ac.uk) and [hkt@berkeley.edu](mailto:hkt@berkeley.edu)

Keywords: Volumetric Additive Manufacturing, Computed Axial Lithography, Controlled polymerization, RAFT

Table of Contents

[S1 – General Rule for RAFT Agent Choice 3](#_Toc221389269)

[S2 - RAFT agent screening through temperature monitoring 5](#_Toc221389270)

[S3 - UV-DSC 7](#_Toc221389271)

[S4 - UV-rheology 10](#_Toc221389272)

[S5 - UV-FTIR 11](#_Toc221389274)

[S6- Thermal imaging setup 12](#_Toc221389275)

[S7 - Scalability of RAFT mediated CAL 13](#_Toc221389276)

[S8 - Results of CAL prints using additional RAFT-incorporating material formulations 17](#_Toc221389277)

[S9 - Printing conditions for CAL prints in Figure 3 19](#_Toc221389278)

[S10 - Printed parts without RAFT 20](#_Toc221389279)

[S11 - Projection optimization for prints shown in Figure 4 21](#_Toc221389280)

[S12 - DSC analysis of post-grafted prints 22](#_Toc221389281)

## S1 – General Rule for RAFT Agent Choice

One of the key features of RAFT polymerization is its ability to polymerize an extensive range of functional monomers, where exceptions mainly include monomers whose functionality may undergo side reactions with the RAFT agent^1,2^. The optimal choice of RAFT agent is inherently monomer-dependent, and screening of different chain-transfer agents is often required to achieve effective control for a given monomer system.

For example, in acrylate-based systems, dithiobenzoate RAFT agents such as CPDB exhibit high chain-transfer efficiency and are therefore particularly effective at suppressing rapid chain growth and associated autoacceleration. For methacrylate systems, dithiobenzoates are expected to undergo slower chain transfer relative to acrylates; however, this is offset by the intrinsically slower propagation kinetics of methacrylates. Because Trommsdorff autoacceleration and the associated heat generation scale with the propagation rate coefficient ($k_{p}$), thermal management during methacrylate polymerization is expected to be comparatively less challenging under RAFT mediation. For other less reactive monomer classes, such as vinyl esters and vinyl amides, RAFT-mediated thermal control is anticipated to be even more straightforward. Suitable RAFT agents are known to facilitate rapid and efficient chain transfer in these systems, enabling suppression of autoacceleration and effective regulation of heat generation during polymerization^1,2^.

The versatility in monomer compatibility of RAFT is related to the reactivity of the CTA, i.e. the C═S bond must be more reactive to radical addition than the monomers C═C bond. This is achieved by careful selection of what are termed the Z- and R- substituents groups within the CTA molecular structure^1,2^. Thus, in considering the choice of RAFT agent for a specific monomer it is important to define the relative reactivity of the CTA and the monomer. Thus, most vinyl monomers can be divided into two categories, either “More “or “Less” activated which is typically related to their electronic stabilization from their substituent, often coupled with steric factors^1,2^. Typically, “more” activated monomer produce relatively stabilized radicals and so require a Z-group that will help with the stabilization of the intermediate radical to favour radical addition on the C═S, e.g., trithiocarbonates or dithiobenzoates. Meanwhile, “less” activated systems are generally less susceptible to homolytic bond scission, and so require the RAFT agent’s intermediate radical to be less stable to favour fragmentation of the propagating radical, e.g. xanthates or dithiocarbamate. The role of the R-group is much more subtle, but it can also influence (1) radical addition to the CTA, (2) subsequent fragmentation from the intermediate formed and (3) propagation. General guidelines for the selection of Z- and R-group can be adapted from previous publications^1,2^.

Our data suggests that the manufacturing method does not fundamentally alter the underlying characteristics of the RAFT mechanism. Accordingly, general guidelines for RAFT-agent selection based on monomer chemistry are expected to remain applicable in CAL/VAM printing. However, these guidelines may require adaptation to reflect the distinct priorities of volumetric additive manufacturing. For example, because CAL/VAM involves crosslinking polymerization, a higher chain-transfer rate than is typically employed in batch solution polymerization of acrylates may be advantageous to delay gelation. Conversely, when thermal moderation is the primary objective, control over radical initialization and re-initialization—often associated with inhibition—may be more critical than achieving strictly linear polymerization kinetics.

## S2 - RAFT agent screening through temperature monitoring


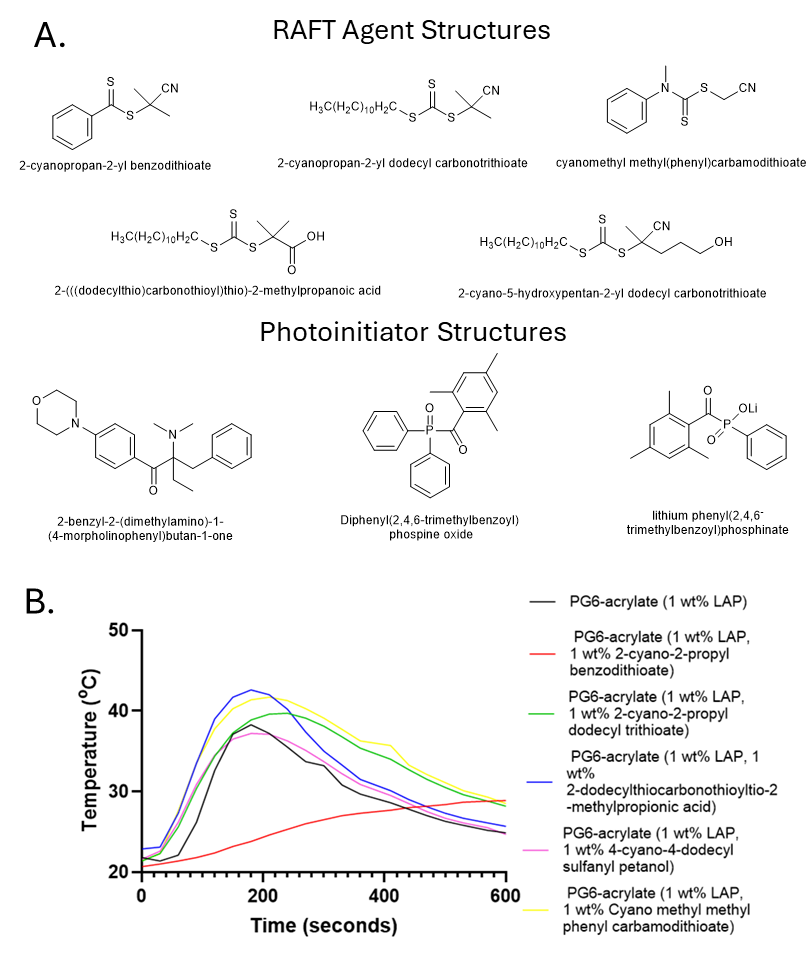


Fig. S1 – RAFT agent screening using PG6-acrylate photopolymerization. A) Molecular structures of raft agents used during screening. B) Temperature evolution during photopolymerization of PG6-acrylate with a variety of RAFT agents.

Fig. S2 – Effect of RAFT agent concentration on temperature generation in PETRA photopolymerization.

Fig. S3 – UV-DSC analysis showing sample temperature of PEGDA photopolymerization utilizing 0.1 wt% LAP. The peak seen in the PEGDA 0.1 wt% LAP curve shows the temperature increase that is caused by the exothermic photopolymerization of this formulation as it is exposed to UV light. In contrast this sharp increase is not seen in the CPBD RAFT agent-containing samples.

## S3 - UV-DSC

Fig. S4 – UV-DSC analysis showing heat flow of PEGDA photopolymerization utilizing 0.1 wt% LAP. The sharp minimum of the PEGDA 0.1 wt% LAP formulation shows that the photopolymerization is rapid and exothermic. In contrast the curves for the CPBD containing formulation show curves with less steep slopes, indicating that the RAFT reactions are not as rapid or as exothermic as the FRP counterpart. Heat flow is plotted such that exothermic events appear as downward peaks.

Fig. S5 – UV-DSC analysis showing sample temperature of PEGDA photopolymerization utilizing 0.4 wt% LAP. The peak seen in the PEGDA 0.4 wt% LAP curve shows the temperature increase that is caused by the exothermic photopolymerization of this formulation as it is exposed to UV light. In contrast this sharp increase is not seen in the CPBD RAFT agent-containing samples.

Fig. S6 – UV-DSC analysis showing heat flow of PEGDA photopolymerization utilizing 0.4 wt% LAP. The sharp minimum of the PEGDA 0.1 wt% LAP formulation shows that the photopolymerization is rapid and exothermic. In contrast the curves for the CPBD-containing formulation show that the curve becomes shallower as increased levels of RAFT agent are added, indicating that the RAFT agents can be used to tune the reactivity and exothermic nature of the formulations. Heat flow is plotted such that exothermic events appear as downward peaks.

Fig. S7 – UV-DSC analysis showing sample temperature of PEGDA photopolymerization utilizing 0.1 wt% LAP and dodecanethiol as a control agent.

Fig. S8 – UV-DSC analysis showing heat flow of PEGDA photopolymerization utilizing 0.1 wt% LAP and dodecanethiol as a control agent. Heat flow is plotted such that exothermic events appear as downward peaks.

## S4 - UV-rheology


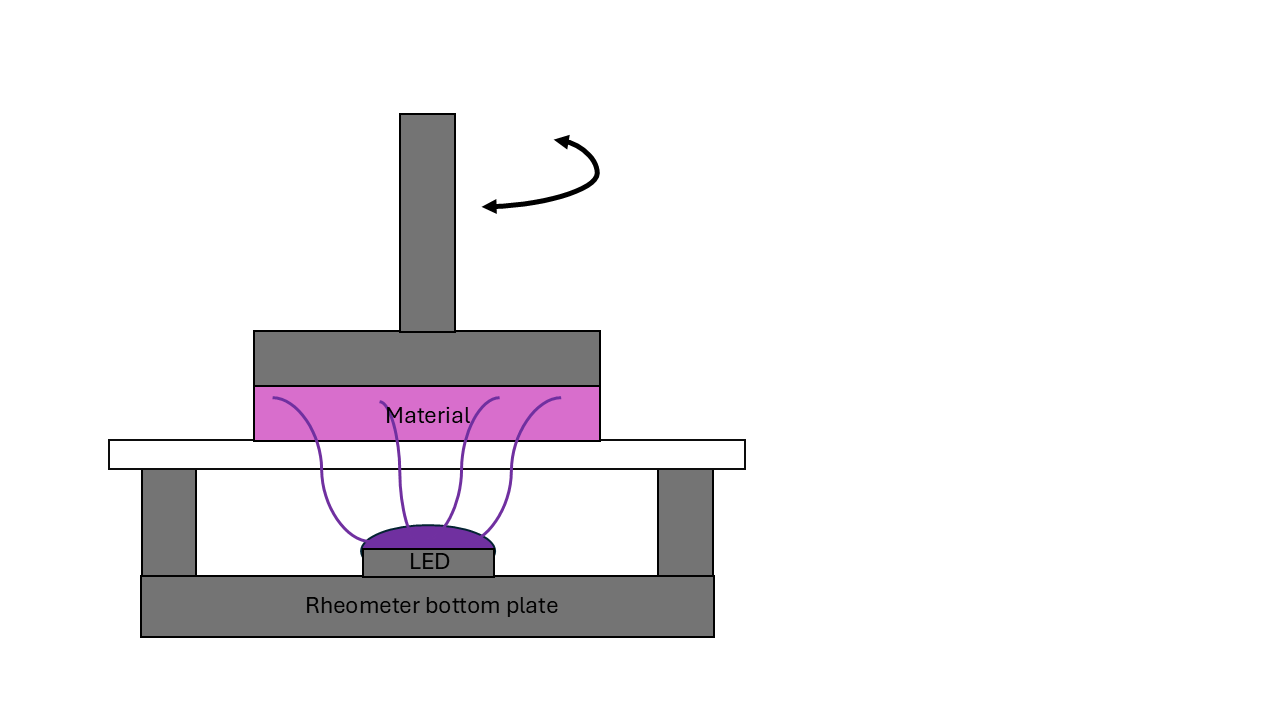


Fig. S9 – UV-rheology setup with customized adaptor


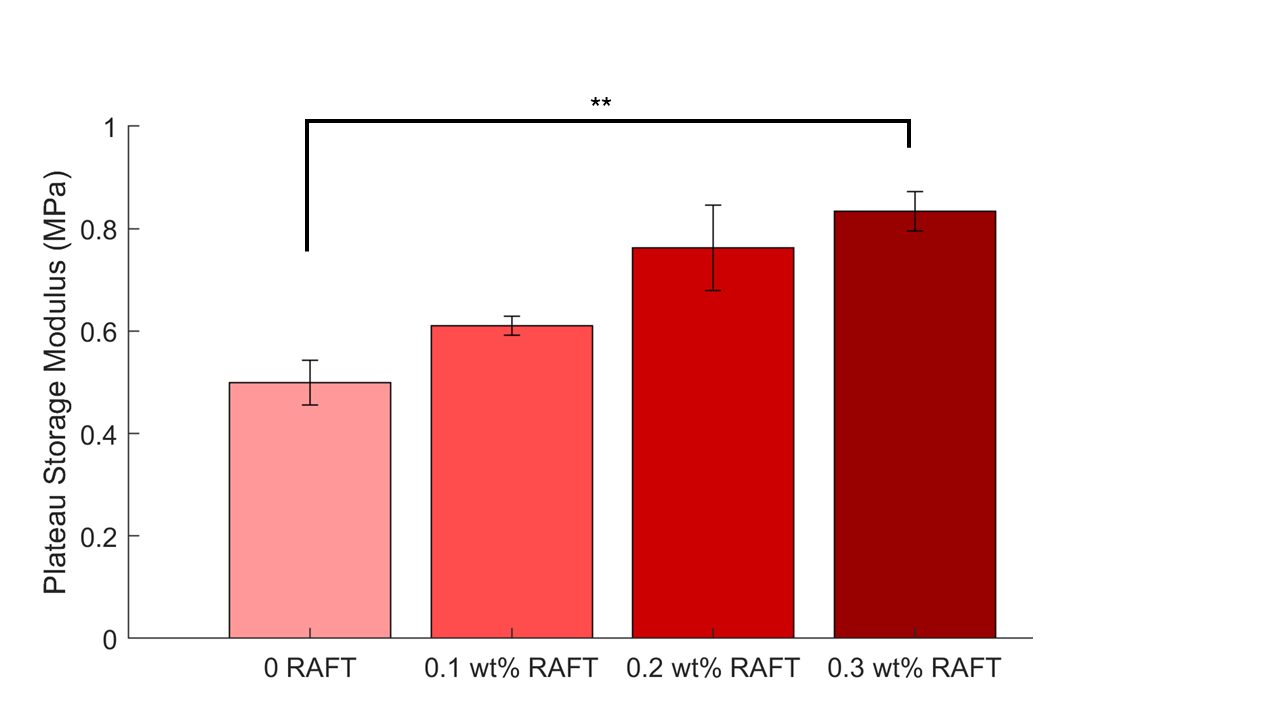


Fig. S10 – Plateau storage modulus of PETRA with 3 mM I369 with and without RAFT measured by UV-rheology. Error bars represent mean standard ± error of the mean (n = 3 for each concentration of RAFT). *p*-values were calculated with one-way ANOVA test. * *p* < 0.05, ** *p* < 0.01.

## S5 - UV-FTIR


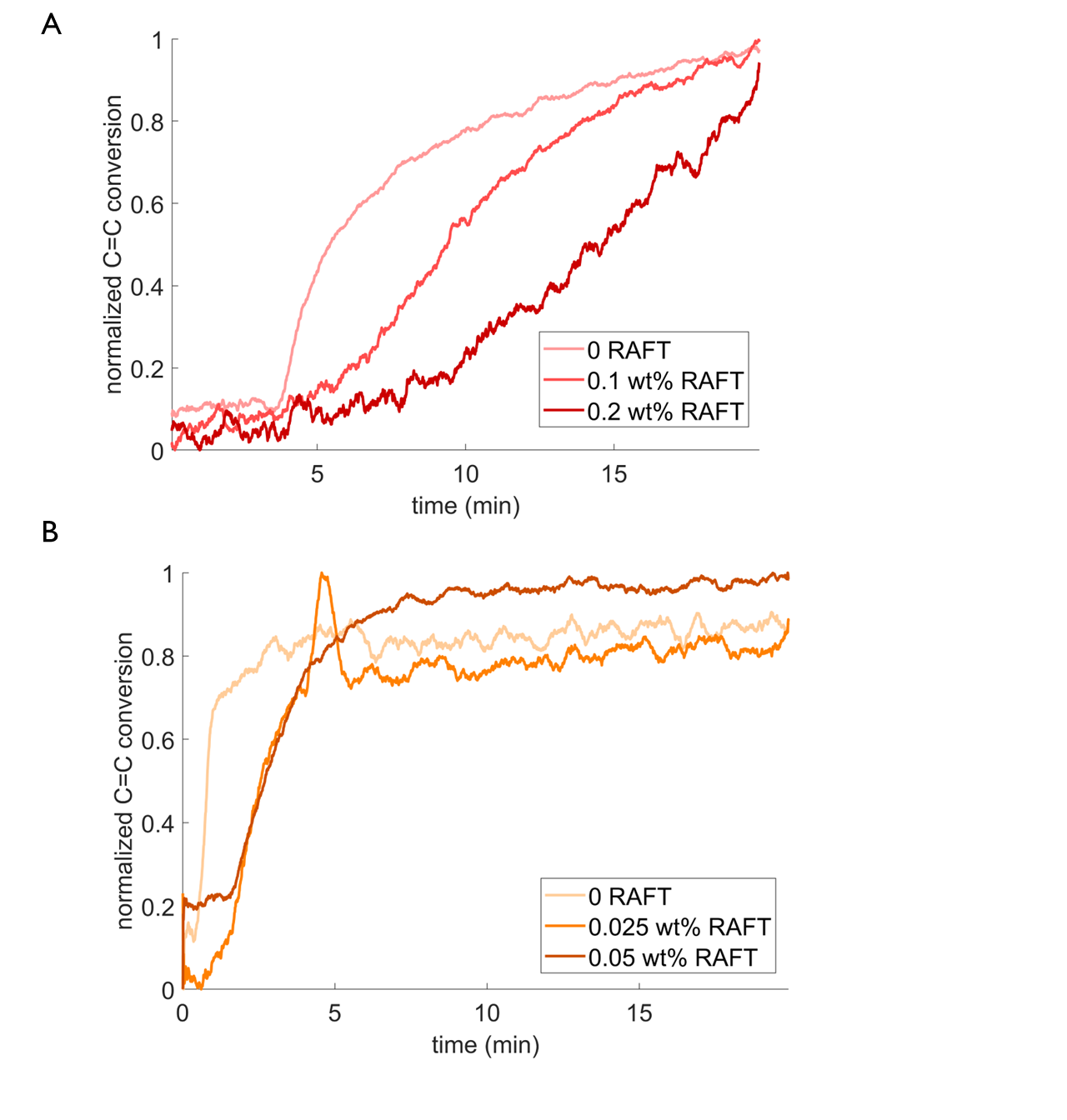


Fig. S11 – Characterization of photopolymerization kinetics via UV-FTIR. Measurement of C=C bond conversion as a function of accumulated illumination time / light dose using UV-FTIR for: A) PETRA with 3 mM I369, comparing conditions without RAFT agent and with 0.1–0.2 wt% RAFT agent (same as Fig. 1A). B) PEG_700_DA:H₂O (80:20 wt%) without RAFT agent and with 0.025–0.05 wt% RAFT agent. Light illumination is turned on at 45 s.

## S6- Thermal imaging setup


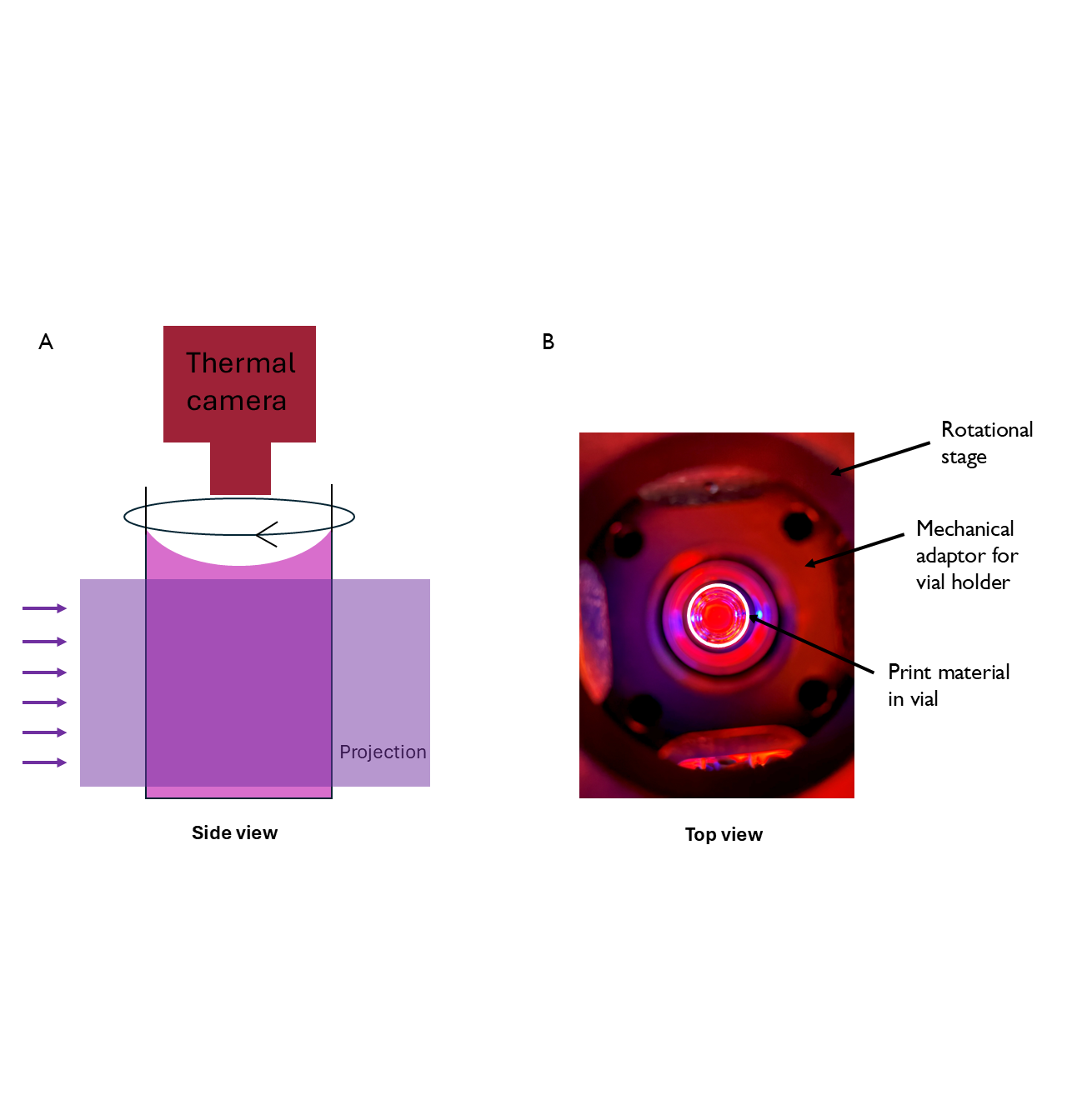


Fig. S12 – Illustration of overhead thermal imaging setup where A). A thermal camera is placed directly above the printing material contained in a glass vial without a cap, and thus the thermal camera does not interfere with the printing process itself and can directly record temperature changes throughout the printing process in real time. B). Top view of the physical setup where the thermal camera is placed above. The thermal camera utilized IR light to measure temperature; therefore the UV light projected for the printing process does not interfere with the measuring and recording of temperature during printing.

## S7 - Scalability of RAFT-mediated CAL

While the present study demonstrates improved thermal stability at the length-scales investigated here (millimeter to centimeter scale), the ultimate scalability of RAFT-mediated CAL will depend on the balance between volumetric heat generation during controlled polymerization and conductive heat dissipation through the build volume. RAFT does not eliminate heat generation; rather, it reduces the instantaneous volumetric heat release by suppressing auto-acceleration and temporarily deactivating propagating radicals through reversible dormancy. Consequently, RAFT-mediated CAL continues to rely on thermal conduction to dissipate reaction heat, but the characteristic length-scale at which conduction becomes insufficient is shifted to substantially larger build volumes relative to uncontrolled free-radical polymerization.

To further assess the scalability of RAFT-mediated thermal control, a three-dimensional transient heat-transfer model was implemented to simulate temperature evolution during CAL printing under conduction-limited conditions. The model solves the transient heat equation assuming isotropic thermal diffusion and volumetric heat generation, with heat transport occurring exclusively through conduction, consistent with the absence of convective mixing in CAL. A single constant thermal diffusivity value ($\alpha=1\times{10}^{-7}\text{ m}\text{2}\text{/s}$) was used in the simulations. This value was estimated for PETRA-based resins based on representative thermophysical properties (viscosity of about 1000 cp) and is consistent with reported thermal-diffusivity ranges for viscous polymeric liquids of comparable rheology^3^; temperature dependence of $\alpha$ was neglected.

The spatial distribution of heat generation was derived from reconstructed three-dimensional light-dose fields obtained via tomographic back-projection of the projected intensity patterns. These dose fields were treated as normalized grayscale volumetric source terms, defining the relative spatial distribution of polymerization-induced heat generation throughout the build volume. Temperature evolution was then computed using a convolution-based Green’s function approach, corresponding to transient heat conduction in an effectively infinite medium.

The transient heat-conduction equation is

$\frac{\partial T(\mathbf{x}, t)}{\partial t}= \alpha\nabla^{2}T\left( \mathbf{x}, t \right)+ q_{T}\left( \mathbf{x}, t \right),$Equation 1

where $T$ is the temperature in Celsius (°C), $\alpha$ is the thermal diffusivity in $\text{m}\text{2}\text{/s}$, and $q_{T}$is the volumetric heat-generation rate expressed in Celsius per second (°C s⁻¹).

For an infinite domain, the temperature field can be written as a spatiotemporal convolution of the heat source with the Green’s function of the diffusion operator:

$T\left( \mathbf{x}, t \right)= \int_{0}^{t} \int_{\mathbb{R}^{3}} G\left( \mathbf{x}-\mathbf{x}^{\boldsymbol{'}}, t- \tau\right){\otimes q}_{T}\left( \mathbf{x}\boldsymbol{'},\tau\right) d\mathbf{x}^{'}d\tau,$ Equation 2

where the Green’s function (heat transfer kernel) is

$G(\mathbf{r},t)=\frac{1}{{(4\pi\alpha t)}^{3/2}}exp(-\frac{\left| \mathbf{r} \right|^{2}}{4\alpha t}), t>0$ Equation 3

and $q_{T}$ can be further expanded and expressed as

$q_{T}\left( \mathbf{x}\boldsymbol{'},\tau\right)=A g\left( \tau\right) W\left( x' \right)$ Equation 4

where $A$ is the maximum magnitude of the effective volumetric heat-generation rate (°C s⁻¹), $g(\tau)$ is a dimensionless temporal gating function that defines when heat generation occurs, accounting for a constant inhibition period before polymerization starts, and $W(\mathbf{x}^{'})$ is the normalized spatial distribution of heat generation derived from the reconstructed three-dimensional dose field.

The absolute magnitude of the volumetric heat-generation rate, expressed in °C s⁻¹, was calibrated using in situ infrared (IR) thermography by fitting the full measured temperature transient to the model response, exploiting the linearity of the governing heat equation with respect to source amplitude. This calibration is necessary because the experimentally measured IR temperature profile reflects the combined effects of heat generation and thermal diffusion and therefore cannot be directly interpreted as the intrinsic volumetric heat-generation rate. Shown in Fig. S13, intrinsic temperature increase per second (A) is obtained by the calibration described using PETRA-based resin with 0.2 wt% RAFT.


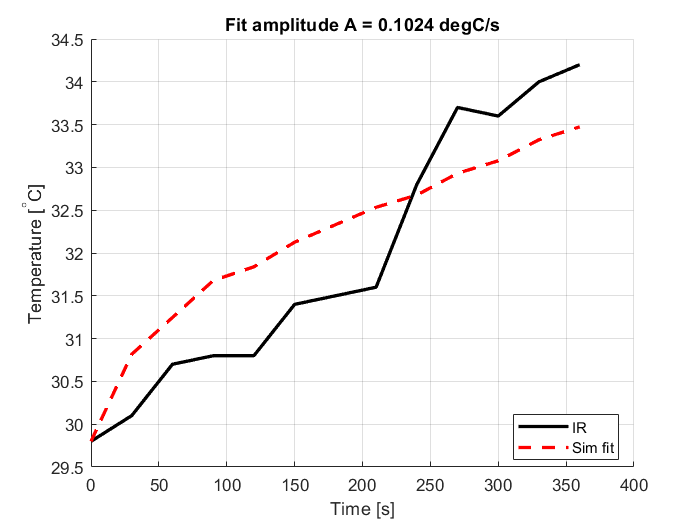


Fig. S13 – Calibration of volumetric heat-generation rate using in situ IR measurement.
Measured infrared (IR) temperature transient during CAL printing (black) and corresponding model prediction (red dashed) obtained by fitting the full temperature history using a convolution-based heat-conduction model. The fitted amplitude $A$ represents the peak effective volumetric heat-generation rate (°C s⁻¹) of PETRA-based resin with 0.2 wt% RAFT, calibrated by exploiting the linearity of the heat equation with respect to source magnitude.

Our model therefore isolates the role of volumetric heat generation and does not account for secondary scaling constraints, such as changes in photoinitiator concentration or printing speed at larger build volumes. In addition, the model does not explicitly simulate autoacceleration phenomena but instead considers only conductive heat dissipation during polymerization. Using this framework, we simulated the maximum temperature rise within a fixed print volume of 0.5 m diameter while printing cylindrical objects with diameters ($D$) ranging from 3 mm to 250 mm, with the cylinder height equal to its diameter. The printing time was fixed at 360 s, corresponding to the exposure duration used to fabricate the cylindrical sample shown in Fig 2. We find that the peak temperature increases plateaus at approximately 17 °C once the cylinder diameter exceeds ~45 mm, as shown in Fig. S14, indicating that beyond this scale the peak temperature is governed primarily by local heat generation rather than overall object size. Such behavior is expected as the characteristic distance ($\mathcal{l}_{d}$) heat can diffuse in time $t$ is $\mathcal{l}_{d}\sim\sqrt{\alpha t}.$ With $\alpha=1\times{10}^{-7}\text{ m}\text{2}\text{/s}$ and t = 360s, $\mathcal{l}_{d}$ is about 6 mm, meaning heat can only diffuse ~6 mm in this printing time. When $\frac{D}{2}$ $\gg$ $\mathcal{l}_{d}$, the peak temperature rise becomes dominated by the local source strength and weakly sensitive to $D$.


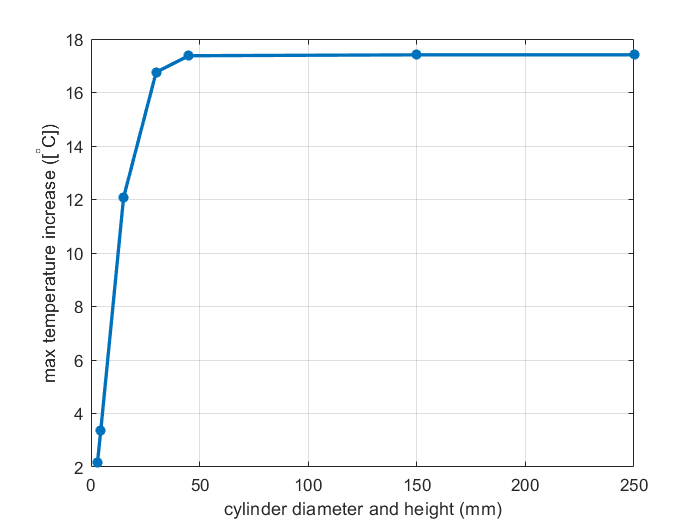


Fig. S14 – Maximum temperature rise as a function of printed cylinder diameter. Simulated peak temperature increase during CAL printing of cylindrical objects with diameters ranging from 3 mm to 250 mm and height equal to diameter, within a fixed 0.5 m print volume. The printing time was fixed at 360 s. The peak temperature increase plateaus beyond ~45 mm diameter, indicating a transition to a conduction-limited regime in which local heat generation dominates over object size.

The next challenge is to scale these experiments to larger print volume. However, as the literature on RAFT generally highlights that rate retardation is an issue with the commercial adoption of RAFT as an industrial technique, the authors do not anticipate significant issues with scaling up RAFT mediated CAL/VAM provided the correct choice of RAFT agent is made for the system being processed

## S8 - Results of CAL prints using additional RAFT-incorporating material formulations


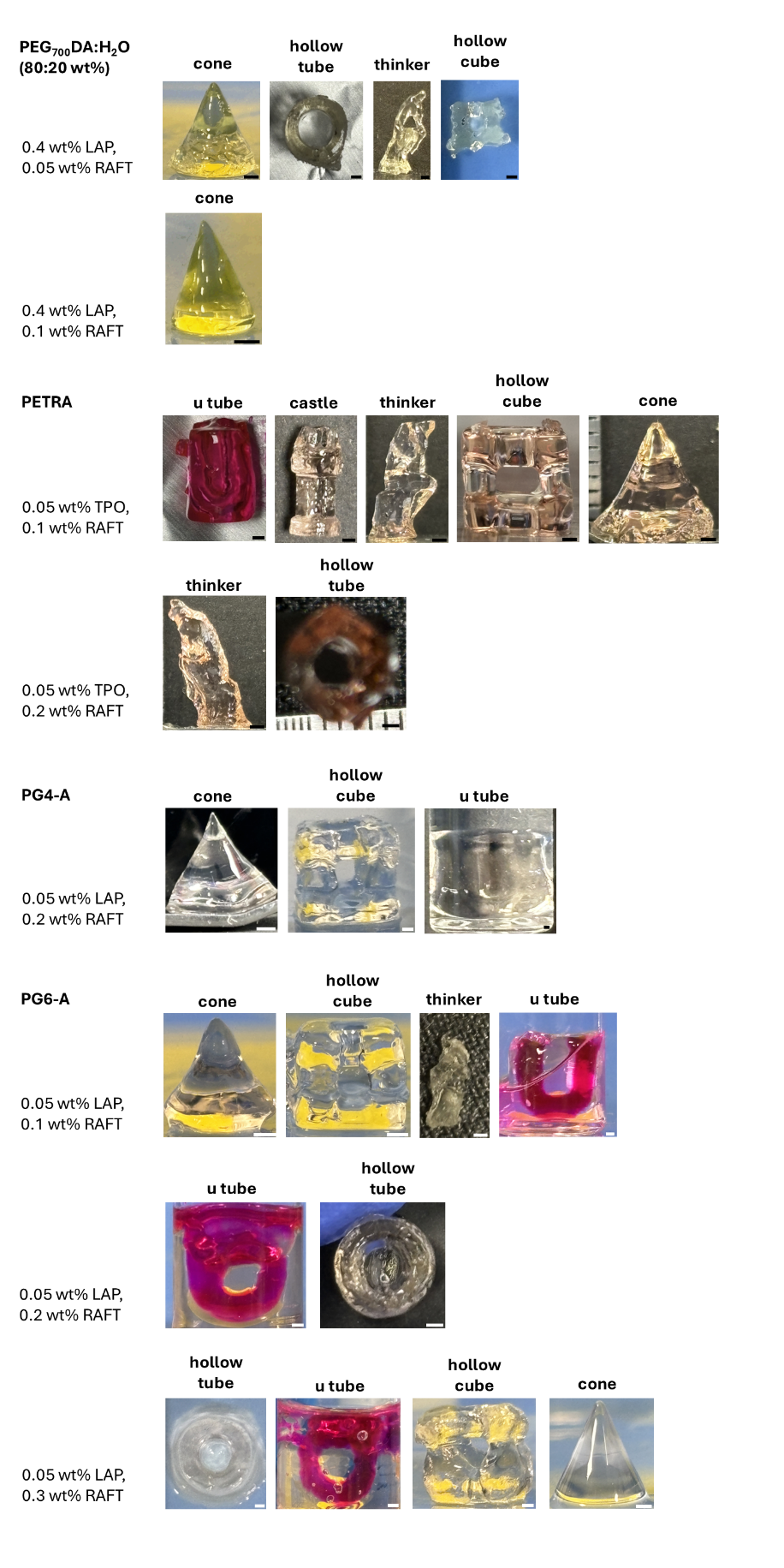


Fig. S15 – printed geometries with additional resin formulations. Scale bars are all 1 mm.

Table S1 Printing conditions of prints in Fig. S15

| Monomer material | Photoinitiator  concentration (wt %) | RAFT concentration (wt %) | model | Intensity scaling factor | Printing time (s) |
| --- | --- | --- | --- | --- | --- |
| PEG_700_DA:H_2_O (80:20 wt%) | LAP: 0.4 | 0.05 | Cone | 1 | 20 |
|  |  |  | Hollow tube | 1 | 60 |
|  |  |  | Thinker | 1 | 30 |
|  |  |  | Hollow cube | 1 | 30 |
|  |  | 0.1 | Cone | 2 | 60 |
| PETRA | TPO: 0.05 | 0.1 | U tube | 1 | 30 |
|  |  |  | Castle | 1 | 45 |
|  |  |  | Thinker | 1 | 35 |
|  |  |  | Hollow cube ^*^ | 1 | 200 |
|  |  |  | Cone | 1 | 45 |
|  |  | 0.2 | Thinker | 2 | 75 |
|  |  |  | Hollow tube | 2 | 75 |
| PG4-A | LAP: 0.05 | 0.2 | Cone | 1 | 240 |
|  |  |  | Hollow cube | 2 | 120 |
|  |  |  | U tube | 2 | 320 |
| PG6-A | LAP: 0.05 | 0.1 | Cone | 1 | 120 |
|  |  |  | Hollow cube | 2 | 130 |
|  |  |  | Thinker | 1 | 380 |
|  |  |  | U tube | 2 | 340 |
|  |  | 0.2 | U tube | 2 | 150 |
|  |  |  | Hollow tube | 2 | 150 |
|  |  | 0.3 | Hollow tube | 2 | 230 |
|  |  |  | U tube | 2 | 195 |
|  |  |  | Hollow cube | 2 | 255 |
|  |  |  | Cone | 2 | 210 |

*printed with the first setup described in Method - CAL printer setup section

Intensity scaling factor is explained in Method - CAL printer setup section

## S9 - Printing conditions for CAL prints in Figure 3

Table S2 Printing conditions of PETRA with 3 mM I369 and 0.1 wt% RAFT

|  | Intensity scaling factor | Printing time (s) |
| --- | --- | --- |
| Thinker | 3 | 75 |
| U-channel | 3 | 90 |
| Y-tube | 6 | 110 |
| Pyramid trusses | 6 | 180 |
| Hollow cube | 3 | 120 |

Table S3. Printing conditions of PEG_700_DA:H_2_O (80:20 wt%) with 0.4 wt% LAP
and 0.025 wt% RAFT

|  | Intensity scaling factor | Printing time (s) |
| --- | --- | --- |
| Thinker | 3 | 30 |
| U-channel | 3 | 35 |
| Y-tube | 3 | 90 |
| Pyramid trusses | 6 | 80 |
| Hollow cube | 3 | 35 |

Table S4. Printing conditions of PG4 with 0.05 wt% LAP and 0.1 wt% RAFT

|  | Intensity scaling factor | Printing time (s) |
| --- | --- | --- |
| Thinker | 5 | 200 |
| U-channel | 3 | 170 |
| Y-tube | 7 | 270 |
| Pyramid trusses | 12 | 240 |
| Hollow cube | 5 | 200 |

## S10 - Printed parts without RAFT


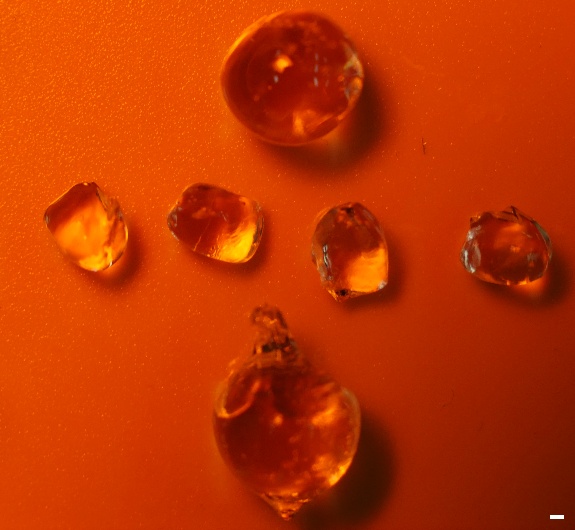


Fig. S16 – Packed spheres with different sizes printed with PETRA containing 3 mM I369 without RAFT. The bottom sphere has additional curing at its top and bottom, which is attributed to the geometry rising due to thermal effect (see SI Movie 4). Scale bar: 1 mm.


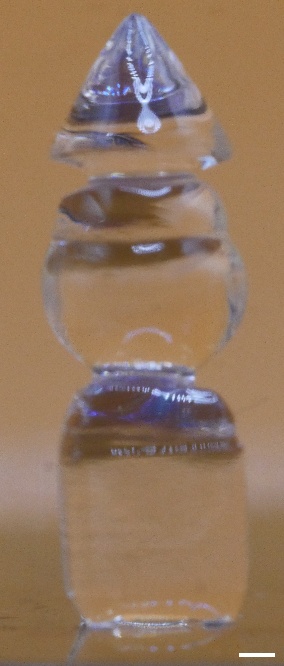


Fig. S17 – Three stacked geometries printed with PETRA containing 3 mM I369 without RAFT. The individual objects are fused together, which is attributed to the rising of parts caused by thermal effects (SI Movie 7). Scale bar: 1 mm.

## S11 - Projection optimization for prints shown in Figure 4

For the print shown in Fig 4B of the main text, an intensity scaling of 1.6 is applied to the *z* slices of projections for the middle spheres. For Fig 4E, greyscale optimization is applied through OSMO with the outermost spherical layer receiving a targeted light dose at 75% of that applied to the inner two layers. The same projection sets are used for both printing with and without RAFT.

## S12 - DSC analysis of post-grafted prints


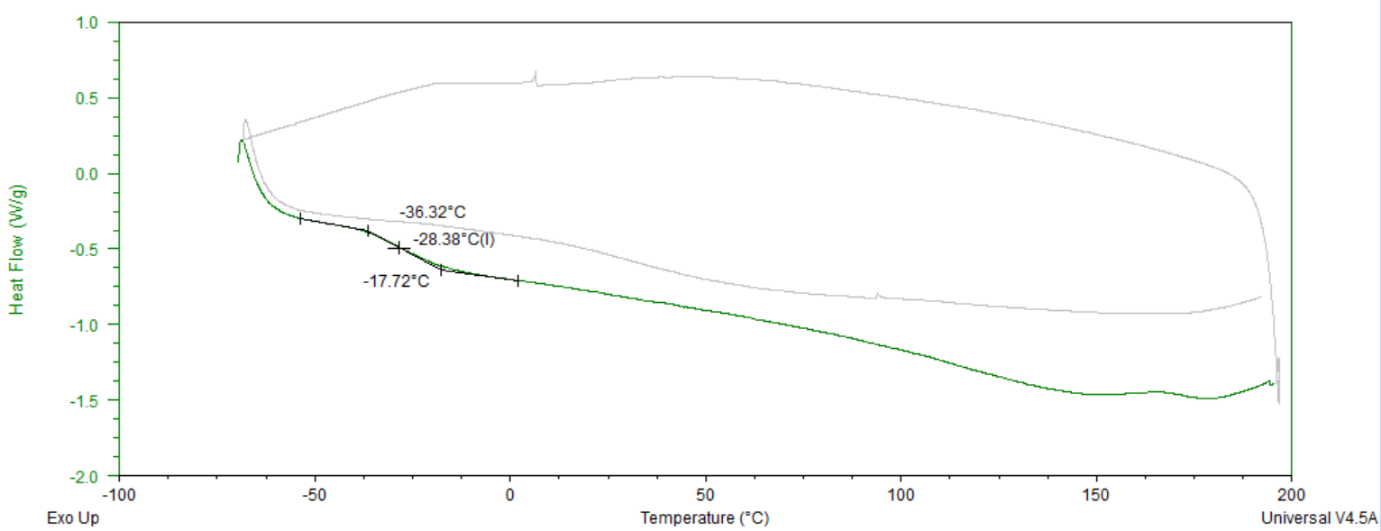


Fig. S18 – DSC analysis of PG6A printed cylinder with 0.3 wt% CPBD. The curve shown is the 1^st^ heating cycle, showing a T_g_ of -28.28⁰C. Heat flow is plotted such that exothermic events appear as downward peaks.


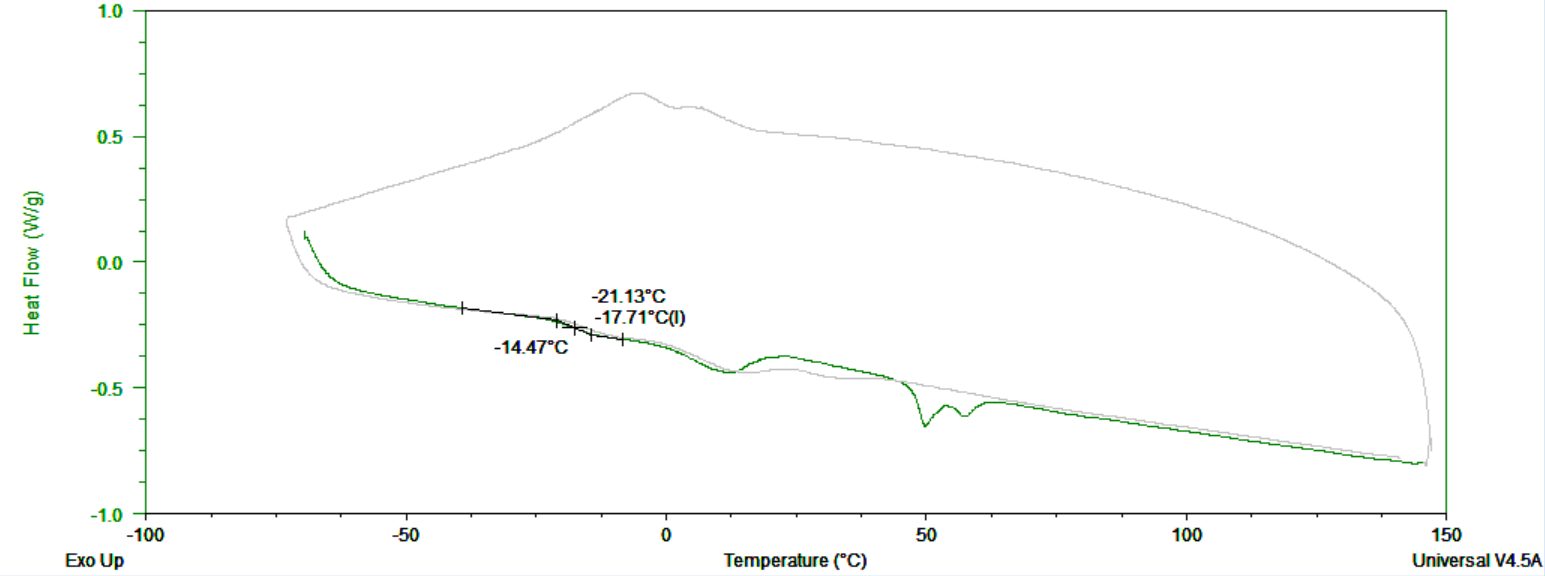


Fig. S19 – DSC analysis of PG6A printed cylinder with 0.3 wt% CPBD with a BA coating. The curve shown is the 1^st^ heating cycle, showing a T_g_ of -17.71⁰C showing that the BA coating has changed the T_g_ of the material. Heat flow is plotted such that exothermic events appear as downward peaks.


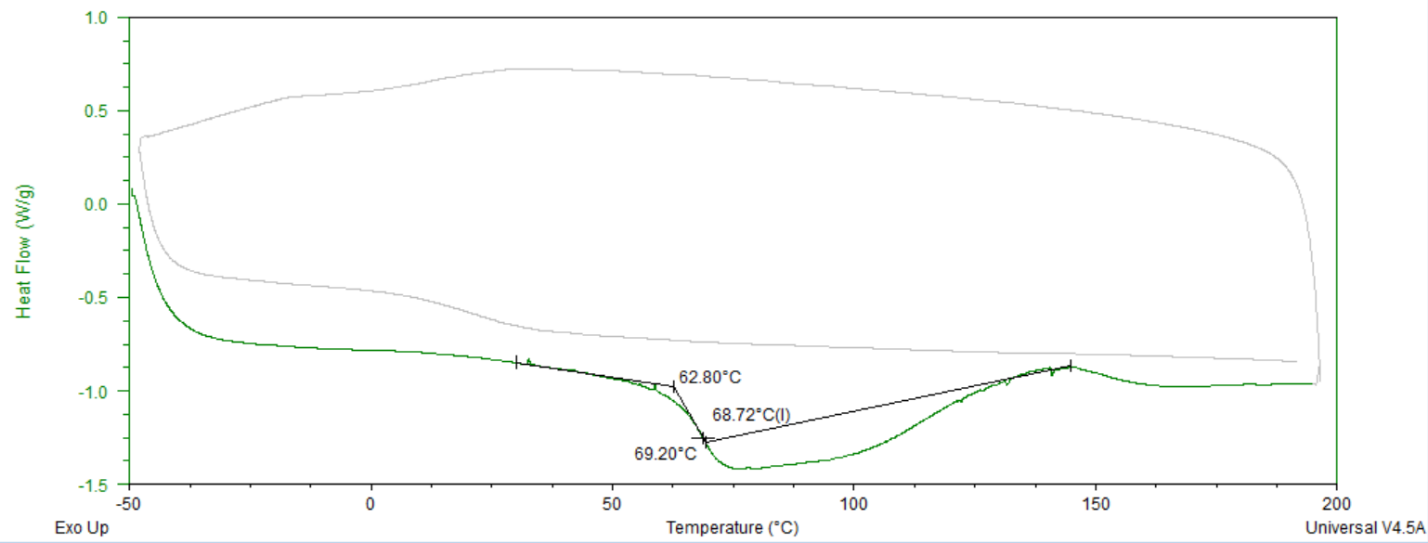


Fig. S20 – DSC analysis of PG6A printed cylinder with 0.3 wt% CPBD with an IBA coating. The curve shown is the 1^st^ heating cycle, showing a T_g_ of -68.72⁰C showing a T_g_ characteristic of IBA. Heat flow is plotted such that exothermic events appear as downward peaks.

Supplementary References:

1. Perrier, S. *50th Anniversary Perspective* : RAFT Polymerization—A User Guide. *Macromolecules* **50**, 7433–7447 (2017).

2. Barner-Kowollik, C. *Handbook of RAFT Polymerization*. (Wiley-VCH, Weinheim, 2008).

3. Spierings, Th. A. M., Bosman, F., Peters, M. C. R. B. & Plasschaert, A. J. M. The thermal diffusivity of two replica resins. *Dental Materials* **4**, 51–54 (1988).
