# Supplementary material for: Enhanced volumetric additive manufacturing via Reversible Addition-Fragmentation Chain Transfer (RAFT) polymerization
Source: Nat Commun. 2026 May 27;17:6907. doi: 10.1038/s41467-026-73456-8 (PMC13389418; doi:10.1038/s41467-026-73456-8)
Supplement: Supplementary file 2 — Description of Additional Supplementary File [file 41467_2026_73456_MOESM2_ESM.pdf]

The Description of Additional Supplementary Files

Supplementary Movie 1: Movie of in-situ monitoring of the FRP-VAM print, showing formation of three spheres and subsequent rising of the parts due to thermal buoyancy.

Supplementary Movie 2: Movie of in-situ monitoring of the RAFT-VAM print, showing formation of three spheres with no rise of parts showcasing successful mitigation of thermal buoyancy during VAM printing.

Supplementary Movie 3: Movie of in-situ monitoring of RAFT-VAM print, showing an increase of volume packing of objects in CAL via printing objects with various feature sizes centered on and away from the rotational axis. Two 10 mm spheres at the top and bottom along the central rotational axis and four 5 mm spheres on separate axes in the middle of the z-axis. The z-axis separation is three pixels.

Supplementary Movie 4: Movie of in-situ monitoring of FRP-VAM print, showing unsuccessful printing of two 10 mm spheres at the top and bottom along the central rotational axis and four 5 mm spheres on separate axes in the middle of the z-axis. The z-axis separation is three pixels.

Supplementary Movie 5: Movie of in-situ monitoring of FRP-VAM print, showing unsuccessful printing of three geometries (a cone, a cube, a sphere) with three-pixel gaps in the z direction.

Supplementary Movie 6: Movie of in-situ monitoring of RAFT-VAM print, showing successful printing of three geometries (a cone, a cube, a sphere) with three-pixel gaps in the z direction.

Supplementary Movie 7: Rotation of hinge structure printed via RAFT-VAM showing that both sides of the hinge could freely rotate around the central pin, showing that RAFT-mediated CAL can be used to print interlocking parts whilst retaining freedom of movement.
