# Supplementary material for: Enhanced volumetric additive manufacturing via Reversible Addition-Fragmentation Chain Transfer (RAFT) polymerization
Source: Nat Commun. 2026 May 27;17:6907. doi: 10.1038/s41467-026-73456-8 (PMC13389418; doi:10.1038/s41467-026-73456-8)
Supplement: Supplementary file 10 — Transparent Peer Review file [file 41467_2026_73456_MOESM10_ESM.pdf]

nature portfolio

Peer Review File

Enhanced Volumetric Additive Manufacturing via Reversible Addition-Fragmentation Chain Transfer (RAFT) Polymerization

Corresponding Author: Professor Derek Irvine

This file contains all reviewer reports in order by version, followed by all author rebuttals in order by version.

Version 0:

Reviewer comments:

Reviewer #1

(Remarks to the Author)

This manuscript introduces RAFT polymerization into Computed Axial Lithography (CAL), enabling much better control over heat generation, reaction kinetics, and buoyancy-driven distortions during volumetric additive manufacturing (VAM). The authors demonstrate suppression of auto-acceleration, more uniform curing, and the ability to print complex, densely packed, multi-scale geometries that cannot be produced using standard free radical polymerization-based CAL. They also show the living character of RAFT end-groups to add post-print surface functionality. Overall, this is a well-designed and compelling piece of work.

I think this is a strong manuscript; however, a few key points should be addressed before acceptance.

Important comment: prior RAFT-mediated 3D printing must be acknowledged

The authors present their work as the first demonstration of RAFT-mediated 3D printing. In my view, their true contribution is the first implementation of RAFT within a CAL/VAM platform, which is genuinely new and impactful. However, RAFT-mediated 3D printing itself has been demonstrated previously (for example by Bagheri et al. and others).

I usually avoid asking authors to cite specific papers, but in this case these prior studies are directly on RAFT-mediated 3D printing and effectively define the existing state of the art in RAFT-based photopolymerization for additive manufacturing. For this reason, it is important that the authors acknowledge the following foundational works:

The first RAFT-based 3D printing systems:

ACS Appl. Polym. Mater. 2020, 2, 782–790

Polym. Chem. 2020, 11, 641–647

Demonstrations of the living character of RAFT-printed materials:

ACS Appl. Polym. Mater. 2022, 4, 4940–4948

Studies on the mechanical impact of RAFT in 3D printing:

ACS Appl. Polym. Mater. 2025, 7, 5846–5854

Applications of RAFT-printing to porous or drug-delivery materials:

ACS Appl. Polym. Mater. 2024, 6, 10853–10864

Acknowledging these prior studies will make it clear that the novelty of this work lies specifically in RAFT-enabled CAL/VAM, rather than RAFT-enabled 3D printing in general, and doing so will make the manuscript both factually accurate and even stronger.

Additional critical questions / points for clarification
1. The study nicely demonstrates improved stability at the scales tested. Can the authors comment on how far they expect this RAFT-based thermal control to scale in terms of build volume and object size before conduction alone becomes insufficient?

2. CPBD is shown to be particularly effective, whereas other RAFT agents are not. To what extent do the authors think their conclusions are general to RAFT vs. specific to high Ctr dithiobenzoates in acrylate systems?

3. A short discussion of how one might select RAFT agents for other monomer classes (e.g. methacrylates, hybrid systems) would be very helpful.

4. The authors mention improved mechanical properties and more homogeneous networks, but the mechanical discussion is relatively brief. Given that RAFT is known to influence network structure and mechanical response, could the authors expand slightly on how the measured mechanical improvements (modulus, toughness, or network homogeneity) compare to RAFT-based 3D printing in the literature?

5. The post-print functionalization relies on retention of RAFT end-groups. Do the authors have any data (or at least expectations) for how stable these end-groups are under typical post-curing, storage, or application conditions? In a previous study, a fluorescence monomer was used to show the livingness of RAFT-based 3D printed materials.

6. The authors mention potential applications such as hydrogel-based drug delivery devices and microreactors. There has been some existing RAFT 3D printing work on drug-delivery scaffolds and porous architectures. Can the authors explicitly articulate what CAL + RAFT uniquely enables relative to layer-by-layer RAFT printing in those contexts?

Reviewer #2

(Remarks to the Author)
This manuscript reported 3D printing using CAL mode. Conventional radical polymerization was compared with reversible addition-fragmentation chain transfer polymerization. The results showed that RAFT polymerization could well suppress the gel effect during CAL 3D printing process, which resulted in good printing resolution. These investigations were interesting and very useful for potentially application of CAL 3D printing in large scale. Thus, it would be potentially of publication for this manuscript in this journal. However, some points need to be considered:
1. The heat accumulation during the polymerization was a complication problem. Many parameters would show effect on the heat accumulation. Some of the parameters have been investigated in this manuscript, such as the polymerization rate etc. I suggested the authors consider the effect of size of items on such heat accumulation. Did different size show effect on the temperature during polymerization?
2. Normally, the addition of RAFT agent will result in more uniform cross linking of the polymer, which will helpful for the elongation of resulted items, however the tensile strength will drop. However, it showed that the tensile strength was enhanced in RAFT way in this manuscript. The authors need to discuss such results. Furthermore, could the authors providing direct results for the effect of RAFT polymerization on the structure of crosslink?
3. Why different colors were observed in different printed items in Figure 4 and Figure 5?
4. Figure 3: why the position of different size balls is different in CRP and RAFT printing?
5. The resolution data of RAFT CAL 3D printing should be provided in the manuscript.

Version 1:

Reviewer comments:

Reviewer #1

(Remarks to the Author)
The authors have adequately addressed the issues raised by the reviewers, and the manuscript is now suitable for publication.

Reviewer #2

(Remarks to the Author)
All of the concerns raised from reviewers were well responded. I supported for the acceptance of this manuscript.

Open Access This Peer Review File is licensed under a Creative Commons Attribution 4.0 International License, which permits use, sharing, adaptation, distribution and reproduction in any medium or format, as long as you give appropriate credit to the original author(s) and the source, provide a link to the Creative Commons license, and indicate if changes were made.
In cases where reviewers are anonymous, credit should be given to 'Anonymous Referee' and the source.
The images or other third party material in this Peer Review File are included in the article’s Creative Commons license, unless indicated otherwise in a credit line to the material. If material is not included in the article’s Creative Commons license and your intended use is not permitted by statutory regulation or exceeds the permitted use, you will need to obtain permission directly from the copyright holder.
To view a copy of this license, visit https://creativecommons.org/licenses/by/4.0/

Response to Reviewer Comments for **Enhanced Volumetric Additive Manufacturing via Reversible Addition-Fragmentation Chain Transfer (RAFT) Polymerization**

Eduards Krumins, Yaxuan Sun, Long Jiang, Vincenzo Taresco, Daniel Keddie, Ricky D. Wildman, Hayden Taylor and Derek Irvine

Reviewer 1

We thank the reviewer for highlighting that this is a strong manuscript. We have addressed this reviewer's comments in the following ways.

1) The authors present their work as the first demonstration of RAFT-mediated 3D printing. In my view, their true contribution is the first implementation of RAFT within a CAL/VAM platform, which is genuinely new and impactful. For this reason, it is important that the authors acknowledge the foundational works listed:

We agree that prior implementation of RAFT in non-VAM 3D printing should be referenced and thank the reviewer for listing these key references, which have duly been incorporated and referred to in the revised text at lines 115-120.

2) The study nicely demonstrates improved stability at the scales tested. Can the authors comment on how far they expect this RAFT-based thermal control to scale in terms of build volume and object size before conduction alone becomes insufficient?

We agree with the reviewer that while the present study demonstrates improved thermal stability at the scales tested (mm to cm scale), the ultimate scalability of RAFT-mediated CAL will depend on the balance between volumetric heat generation of controlled polymerization and conductive heat dissipation through the build volume. Ongoing work is therefore focused on experimentally validating this scaling behaviour using substantially larger print volumes enabled by a customized, large-scale CAL platform. While these results are not yet available for inclusion in the present manuscript, important insights can be drawn from prior literature on RAFT polymerization.

Specifically, RAFT polymerization is widely reported to exhibit reduced instantaneous polymerization rates relative to uncontrolled free-radical polymerization at large scale due to reversible radical deactivation and the presence of dormant chain states. Furthermore, this rate retardation can be exacerbated when an incorrect chain-transfer agent:monomer system combination is utilised [1]*. Consequently, this is a factor that has historically limited the industrial adoption of RAFT, and much research has been conducted to try to reduce/remove this slowing of the polymer production rate.*

However, the focus of this project is different from most RAFT-related research, as it is endeavouring to utilise the RAFT-induced reaction retardation and its fundamental reduction in peak heat-generation rate [2,3]*, rather than removing it. Thus, in the same way that the RAFT production of a specific molecular structure can be optimised by choosing the correct RAFT agent/monomer combination, initiator concentration and the level of monomer usage by applying the published protocols*[1]*. So, the rate of retardation will also be predictable by applying the same protocols to define that the appropriate RAFT agent is selected for the resin chemistry, with this thermal control as a differentiated goal. Consequently, RAFT-mediated CAL is not expected to face the intrinsic limitations associated with conduction-limited heat removal during scale-up. Rather, the benefits of*

applying RAFT chemistry to VAM will be a general concept that can be linked to pre-screening of proposed VAM resins chemistry via photo DSC (S3).

To further support this expectation, we have added a section entitled "Scalability of RAFT mediated CAL" (S7) to the Supplementary Information, which provides additional discussion and a physics-based thermal scaling analysis examining the interplay between RAFT-regulated heat generation and conductive heat dissipation as a function of build size. Specifically, we include transient heat-conduction simulations using thermal diffusivity values estimated for representative PETRA formulations based on resin viscosity, coupled with volumetric heat-generation terms calibrated from in situ infrared temperature measurements. The model captures the competition between volumetric heat generation during photopolymerization and heat dissipation by conduction. Our model predicts that once the object size exceeds the characteristic thermal diffusion length, which is governed by the printing time and the thermal diffusivity, the peak temperature rise becomes dominated by the local volumetric heat-generation rate and only weakly dependent on the overall object scale. Using the volumetric heat-generation rate calibrated from in situ IR thermography for the RAFT-containing formulation ($A = 0.1024$ °C s^{-1}) and a fixed printing time of 360 s, the simulated maximum temperature rise, including the effects of thermal diffusion, plateaus at approximately 17 °C. This analysis implicitly assumes that RAFT-mediated polymerization effectively suppresses the Trommsdorff (autoacceleration) effect, an assumption that is consistent with prior RAFT polymerization literature. We noted that this model needs to be further verified by experimental results as it does not account for secondary scaling constraints, such as changes in photoinitiator concentration or printing speed at larger build volume.

To introduce this new discussion the following text has been added to the main text "*Furthermore, to support the proposition that RAFT mediated VAM can be scaled successfully, a physics-based thermal scaling analysis was performed using calibrated data from the in-situ infrared temperature measurements made during our experimental program for representative PETRA formulations. This model predicted that once the object size exceeds the characteristic thermal diffusion length, governed by the printing time and the thermal diffusivity, the peak temperature rise becomes dominated by the local volumetric heat-generation rate and was only weakly dependent on the overall object scale. See detailed discussion of simulations conducted in discussion S7 which includes Figs. S13 and S14." (lines 230-237).*

References:

1. *Nwoko, T.; Nguyen, K.; Saha, N. K.; Barner-Kowollik, C.; Konkolewicz, D. Rate Retardation Trends in RAFT – an Emerging Monomer Classification Tool? Polym. Chem.* ***2024****, 15 (11), 1052–1061.* https://doi.org/10.1039/D3PY01332D.
2. *Perrier, S. 50th Anniversary Perspective : RAFT Polymerization—A User Guide. Macromolecules 50, 7433–7447 (2017).*
3. *Barner-Kowollik, C. Handbook of RAFT Polymerization. Wiley-VCH, Weinheim (2008)*

3) CPBD is shown to be particularly effective, whereas other RAFT agents are not. To what extent do the authors think their conclusions are general to RAFT vs. specific to high Ctr dithiobenzoates in acrylate systems?

We believe that our conclusions will be general to RAFT chemistry as the retardation can be influenced by RAFT agent:monomer combinations, and by managing initiator concentration and the level of monomer usage.

From the authors' perspective, dithiobenzoate RAFT agents exhibit different transfer behaviors depending on monomer class. For example, chain transfer is typically slower during methacrylate polymerization compared with acrylate systems. However, from a thermal management standpoint, these differences are partially offset by the intrinsically slower kinetics of methacrylate polymerization. Consequently, thermal control during methacrylate-based printing is expected to be comparatively less challenging. This arises because Trommsdorff (auto-acceleration) effects and associated heat generation depend strongly on the propagation rate coefficient (k_p), which is lower for methacrylates. For less active monomers such as vinyl esters and vinyl amides, thermal management is expected to be even more straightforward, as suitable RAFT agents are known to facilitate efficient chain transfer during polymerization of these systems.

In this study, we intentionally adopted a no-preconception strategy for RAFT agent selection, rather than restricting our investigation to agents traditionally paired with monomers used in VAM printing. This approach was chosen to directly test whether the manufacturing process fundamentally alters the behavior of the RAFT mechanism. Our results indicate that it does not; rather, RAFT agent selection remains a critical determinant of performance, and the general characteristics of RAFT polymerization are preserved under CAL/VAM conditions. To clarify this point, we have added additional discussion to Supplementary Information Section S1. The relevant passage states: "Our data suggests that the manufacturing method does not fundamentally alter the underlying characteristics of the RAFT mechanism. Accordingly, general guidelines for RAFT-agent selection based on monomer chemistry are expected to remain applicable in CAL/VAM printing.." (lines 254-257).

4) A short discussion of how one might select RAFT agents for other monomer classes (e.g. methacrylates, hybrid systems) would be very helpful.

To address this comment, we have added two sections of text to documents. One in the main manuscript and a second to the supporting information document. The section in the main text is designed to highlight the point that the reviewer has made above. However, as we did not want the main manuscript becoming too lengthy, we have added a longer discussion on key items that should be considered when choosing RAFT Agents into the Supporting Document text, should readers want to read further.

The text added in the main manuscript reads:
(a)" One of the key features of RAFT polymerization is its ability to control the polymerization of a wide range of functional monomers[28,41]. However, the monomer compatibility of RAFT is strongly influenced by the reactivity of the chain-transfer agent (CTA), and careful selection of the CTA molecular structure is required to achieve optimal control in conventional solution-based RAFT polymerization[28,41]. As a result, a set of established general rules exists to guide the selection of appropriate RAFT agents for

specific monomers or monomer combinations. A brief discussion of these solution-based RAFT guidelines is provided in the Supporting Information (S1)." Lines (157-163)

(b) "Our data suggest that the manufacturing method does not fundamentally alter the underlying characteristics of the RAFT mechanism. Accordingly, general guidelines for RAFT-agent selection based on monomer chemistry are expected to remain applicable in CAL/VAM printing." (lines 254-257).

The Discussion in S1 reads:
"One of the key features of RAFT polymerization is its ability to polymerize an extensive range of functional monomers, where exceptions mainly include monomers whose functionality may undergo side reactions with the RAFT agent[1,2]. The optimal choice of RAFT agent is inherently monomer-dependent, and screening of different chain-transfer agents is often required to achieve effective control for a given monomer system.

For example, in acrylate-based systems, dithiobenzoate RAFT agents such as CPDB exhibit high chain-transfer efficiency and are therefore particularly effective at suppressing rapid chain growth and associated autoacceleration. For methacrylate systems, dithiobenzoates are expected to undergo slower chain transfer relative to acrylates; however, this is offset by the intrinsically slower propagation kinetics of methacrylates. Because Trommsdorff autoacceleration and the associated heat generation scale with the propagation rate coefficient (k_p), thermal management during methacrylate polymerization is expected to be comparatively less challenging under RAFT mediation. For other less reactive monomer classes, such as vinyl esters and vinyl amides, RAFT-mediated thermal control is anticipated to be even more straightforward. Suitable RAFT agents are known to facilitate rapid and efficient chain transfer in these systems, enabling suppression of autoacceleration and effective regulation of heat generation during polymerization[1,2].

The versatility in monomer compatibility of RAFT is related to the reactivity of the CTA, i.e. the C═S bond must be more reactive to radical addition than the monomers C═C bond. This is achieved by careful selection of what are termed the Z- and R- substituents groups within the CTA molecular structure[1,2]. Thus, in considering the choice of RAFT agent for a specific monomer it is important to define the relative reactivity of the CTA and the monomer. Thus, most vinyl monomers can be divided into two categories, either "More "or "Less" activated which is typically related to their electronic stabilization from their substituent, often coupled with steric factors[1,2]. Typically, "more" activated monomer produce relatively stabilized radicals and so require a Z-group that will help with the stabilization of the intermediate radical to favour radical addition on the C═S, e.g., trithiocarbonates or dithiobenzoates. Meanwhile, "less" activated systems are generally less susceptible to homolytic bond scission, and so require the RAFT agent's intermediate radical to be less stable to favour fragmentation of the propagating radical, e.g. xanthates or dithiocarbamate. The role of the R-group is much more subtle, but it can also influence (1) radical addition to the CTA, (2) subsequent fragmentation from the intermediate formed and (3) propagation. General guidelines for the selection of Z- and R-group can be adapted from previous publications[1,2].

Our data suggests that the manufacturing method does not fundamentally alter the underlying characteristics of the RAFT mechanism. Accordingly, general guidelines for RAFT-agent selection based on monomer chemistry are expected to remain applicable in

CAL/VAM printing. However, these guidelines may require adaptation to reflect the distinct priorities of volumetric additive manufacturing. For example, because CAL/VAM involves crosslinking polymerization, a higher chain-transfer rate than is typically employed in batch solution polymerization of acrylates may be advantageous to delay gelation. Conversely, when thermal moderation is the primary objective, control over radical initialization and re-initialization—often associated with inhibition—may be more critical than achieving strictly linear polymerization kinetics."

References:

1. *Perrier, S. 50th Anniversary Perspective : RAFT Polymerization—A User Guide. Macromolecules **50**, 7433–7447 (2017).*
2. *Barner-Kowollik, C. Handbook of RAFT Polymerization. (Wiley-VCH, Weinheim, 2008).*
3. *Spierings, Th. A. M., Bosman, F., Peters, M. C. R. B. & Plasschaert, A. J. M. The thermal diffusivity of two replica resins. Dental Materials **4**, 51–54 (1988).*

5) The authors mention improved mechanical properties and more homogeneous networks, but the mechanical discussion is relatively brief. Given that RAFT is known to influence network structure and mechanical response, could the authors expand slightly on how the measured mechanical improvements (modulus, toughness, or network homogeneity) compare to RAFT-based 3D printing in the literature?

We thank the reviewer for this question, which allows us to give a deeper explanation about the mechanical property differences that are seen and are important to emphasize in our study. The differences in mechanical strength between the FRP and RAFT-derived polymers can be rationalized in terms of differences in specific free volume arising from polymerization kinetics. Rapid free-radical polymerization is known to produce glassy polymer networks that are kinetically trapped in non-equilibrium states, resulting in higher free volume and increased microstructural heterogeneity. Cure-rate-dependent studies on thermoset polymers demonstrate that the rate of network formation directly influences the size and distribution of free-volume holes in the final polymer, even when the degree of cure and glass transition temperature are comparable, indicating that local packing is governed by kinetics rather than conversion alone[1]. Experimental investigations using positron annihilation lifetime spectroscopy further show that variations in free-volume hole size and fraction correlate with changes in mechanical properties such as modulus and strength in epoxy networks[2]. Physical aging studies similarly identify free volume as a key structural parameter controlling the mechanical response of amorphous polymer networks, with reductions in free volume leading to measurable changes in stiffness and viscoelastic behaviour[3].

Incorporation of RAFT agents retards the polymerization process, reducing kinetic trapping during network formation and leading to a more efficiently packed polymer network with a lower and more homogeneous free-volume distribution. [1] Such reductions in free volume are consistent with reports linking improved packing efficiency to enhanced mechanical performance in thermoset polymers[2,3]. This trend is reflected in the increasing storage modulus with increasing RAFT concentration, as measured by UV rheology (Fig. 2A and Fig. S10). As RAFT agent concentration increases, the influence of kinetic control on free-volume reduction is expected to diminish once a limiting packing state is reached, resulting in a plateau in mechanical properties. Differences in free-volume distribution may also contribute to the refractive-index variations observed between FRP and RAFT-printed

materials in shadowgraph images, as refractive index is sensitive to density fluctuations and local heterogeneity within polymer networks. As shown in Fig. 2D and Fig. 3, prints produced with RAFT-containing resins appear less optically visible in shadowgraph, indicating a smaller refractive-index (RI) change[4]. As stated in the main manuscript (lines 268 - 269), this smaller refractive-index change during printing likely contributed to the smoother surface finish observed in RAFT-based prints. This ability to influence free volume through polymerization kinetics therefore suggests a potential route for tuning optical properties, which may be advantageous for large-scale printing applications in terms of surface finishing and will be explored in future work.

Thus, the following text has been added to the main manuscript to expand our discussion as requested "Incorporation of RAFT agents retards the polymerization process, reducing kinetic trapping during network formation. This leads to a more efficiently packed polymer network with a lower and more homogeneous free-volume distribution and has been linked to enhanced mechanical performance in thermoset polymers[43]. Furthermore, as RAFT agent concentration increases, the influence of kinetic control on free-volume reduction is expected to diminish once a limiting packing state is reached, resulting in a plateau in mechanical properties observed in the data[44,45]." (lines 212-218)

References

1. *Childers, C. H., Hassan, M. K., Mauritz, K. A. & Wiggins, J. S. Molecular scale cure rate dependence of thermoset matrix polymers. Arab. J. Chem. 9, 206–218 (2016).*
2. *Zhang, H. J. et al. Effect of free-volume holes on mechanical properties of epoxy resins studied by positron annihilation and PVT experiments. Polymer 98, 47–55 (2016).*
3. *Odegard, G. M. & Bandyopadhyay, A. Physical aging of epoxy polymers and their composites. J. Polym. Sci. B: Polym. Phys. 49, 1695–1716 (2011).*
4. *Chung Li, C., Toombs, J. & Taylor, H. Tomographic color Schlieren refractive index mapping for computed axial lithography. in Symposium on Computational Fabrication 1–7 (ACM, Virtual Event USA, 2020). doi:10.1145/3424630.3425421.*

6) The post-print functionalization relies on retention of RAFT end-groups. Do the authors have any data (or at least expectations) for how stable these end-groups are under typical post-curing, storage, or application conditions? In a previous study, a fluorescence monomer was used to show the livingness of RAFT-based 3D printed materials.

At this stage, we do not yet have a systematic understanding of the stability of RAFT end-groups under post-curing, storage, or application-relevant conditions. Existing literature on RAFT-based 3D printed materials suggests that RAFT end-groups can remain sufficiently intact to enable post-polymerization and functionalization, as demonstrated, for example, by surface-initiated regrowth using fluorescent monomers. However, the efficiency of such regrowth is known to be sensitive to environmental factors such as oxygen exposure, temperature, and processing history.

In the present work, post-functionalization was therefore limited to a proof-of-concept demonstration that polymerization from retained RAFT end-groups is possible. A more comprehensive investigation of end-group stability and regrowth efficiency under controlled post-curing and storage conditions is the subject of ongoing and future work. To illustrate this point in the main manuscript, we added "However, we note that stable post-print

functionalization under various post-curing, storage, or application conditions will require further optimization of processing conditions" at lines 421-422.

7) The authors mention potential applications such as hydrogel-based drug delivery devices and microreactors. There has been some existing RAFT 3D printing work on drug-delivery scaffolds and porous architectures. Can the authors explicitly articulate what CAL + RAFT uniquely enables relative to layer-by-layer RAFT printing in those contexts?

   CAL's advantages over conventional layer-by-layer printing techniques, as outlined in the Introduction (line 41-53), remain preserved in RAFT-mediated CAL. These include rapid fabrication, i.e. on the order of ~1 min for centimeter-scale objects, support-free printing, reduced post-processing and material waste, the ability to fabricate highly complex geometries, and improved surface finish that mitigates inter-layer delamination. Importantly, these benefits are not achievable with layer-by-layer RAFT printing approaches and are particularly advantageous for applications, such as hydrogel-based drug delivery devices and microreactors, where continuous, smooth internal architectures, rapid fabrication, and intricate geometries are critical.

   Beyond preserving the intrinsic advantages of CAL, the incorporation of RAFT chemistry uniquely enhances the CAL process itself. RAFT-mediated CAL improves resin stability during volumetric printing, enabling more reliable fabrication and facilitating scale-up to larger build volumes. In addition, the living character of the RAFT mechanism enables post-printing chemical modification and multi-material functionalization. Collectively, CAL + RAFT expands both the manufacturability and functional versatility of volumetrically printed devices for applications listed in the manuscript.

   Thus, we have added the following text to highlight these aspects as requested by the reviewer, "This is because the benefits of conventional CAL over layer-by-layer printing approaches, e.g. ultrafast printing speed, freedom of geometry, and mitigation of mechanical weaknesses associated with inter-layer delamination, are retained in RAFT-mediated CAL. Furthermore, the improved resin stability and the presence of surface located active RAFT headgroups could lead to the 4D-printing of actuators capable of dynamic shape transformation via post-grafting" in lines 440-445.

Reviewer 2

We thank the reviewer for highlighting that our investigations are interesting and very useful for potentially application of CAL 3D printing in large scale.

1) The heat accumulation during the polymerization was a complication problem. Many parameters would show effect on the heat accumulation. Some of the parameters have been investigated in this manuscript, such as the polymerization rate etc. I suggested the authors consider the effect of size of items on such heat accumulation. Did different size show effect on the temperature during polymerization?

   We thank the reviewer for raising this important point. We agree with the reviewer that object size can influence heat accumulation during photopolymerization. Thus, in our experiments, we directly probed size effects by printing multiple objects of different

diameters, ranging from 2.5 mm to 10 mm, simultaneously within the same vat (shown in Figure 3 and discussed in main manuscript lines 258 to 268). In the non-RAFT case, larger objects exhibited increased heat accumulation, leading to elevated local temperatures that generated buoyancy forces in the resin. This caused partially cured structures to rise prematurely within the vat, resulting in merging of adjacent structures. In contrast, under RAFT-mediated conditions, objects across this size range remained structurally independent and faithful to the design geometry, indicating that heat accumulation was effectively mitigated. In other words, any temperature rise did not increase to a level that induced overcuring and/or buoyance-driven instabilities as object size increased. Thus, direct in-situ temperature measurements were not possible for each individual object size without disturbing the vat. The observed suppression of overcuring and thermal crosstalk are compelling indirect evidence that RAFT-mediated CAL reduces size-dependent heat accumulation effects relative to uncontrolled free-radical polymerization over the millimeter to centimeter-scale range investigated. Consequently, a detailed investigation of size-dependent thermal behavior at larger length scales is the subject of ongoing work.

Although these results are not yet available for inclusion in the present manuscript, we have added Section S7 to Supplementary Information, which presents a physics-based scaling analysis for our model RAFT-containing resin and examines how feature size affects heat accumulation within a conduction-limited framework. This analysis predicts that once the feature size exceeds the characteristic thermal diffusion length, which is determined by the printing time and thermal diffusivity, the peak temperature rise becomes dominated by local volumetric heat generation and only weakly dependent on the overall object size. This interpretation assumed that RAFT-mediated polymerization effectively suppresses the Trommsdorff (i.e. auto-acceleration) effect, consistent with prior RAFT polymerization literature [1,2]. By contrast, for uncontrolled free-radical polymerization, such assumptions do not hold, and a comparable assessment of size-dependent heat accumulation will require direct experimental validation, which is also the focus of ongoing work.

Reference:

1. Perrier, S. *50th Anniversary Perspective*: RAFT Polymerization—A User Guide. *Macromolecules* 50, 7433–7447 (2017).
2. Barner-Kowollik, C. *Handbook of RAFT Polymerization.* Wiley-VCH, Weinheim (2008)

2) Normally, the addition of RAFT agent will result in more uniform cross linking of the polymer, which will helpful for the elongation of resulted items, however the tensile strength will drop. However, it showed that the tensile strength was enhanced in RAFT way in this manuscript. The authors need to discuss such results. Furthermore, could the authors providing direct results for the effect of RAFT polymerization on the structure of crosslink?

We thank the reviewer for this insightful comment. We note that tensile strength was not directly measured in this study; therefore, our discussion focuses on rheological and structural indicators that suggest changes in network mechanical behaviour rather than

direct tensile performance. While RAFT polymerization can in some cases reduce tensile strength due to decreased crosslink density or slower polymerization kinetics, our UV-rheology measurements showed an increase in storage modulus with increasing RAFT concentration (Fig. 2A and Fig. S10), indicating enhanced network stiffness.

We have rationalised the differences in mechanical strength between the FRP and RAFT-derived polymers in terms of differences in specific free volume arising from the polymerization kinetics. Rapid free-radical polymerization is known to produce glassy polymer networks that are kinetically trapped in non-equilibrium states, resulting in higher free volume and increased microstructural heterogeneity. Cure-rate-dependent studies on thermoset polymers demonstrate that the rate of network formation directly influences the size and distribution of free-volume holes in the final polymer, even when the degree of cure and glass transition temperature are comparable, indicating that local packing is governed by kinetics rather than conversion alone[1]. Experimental investigations using positron annihilation lifetime spectroscopy further show that variations in free-volume hole size and fraction correlate with changes in mechanical properties such as modulus and strength in epoxy networks[2]. Physical aging studies similarly identify free volume as a key structural parameter controlling the mechanical response of amorphous polymer networks, with reductions in free volume leading to measurable changes in stiffness and viscoelastic behaviour[3].

Incorporation of RAFT agents slightly retards the polymerization process, reducing kinetic trapping during network formation and leading to a more efficiently packed polymer network with a lower and more homogeneous free-volume distribution. Such reductions in free volume are consistent with reports linking improved packing efficiency to enhanced mechanical performance in thermoset polymers[2,3]. This trend is reflected in increasing storage modulus with increasing RAFT concentration, as measured by UV rheology (Fig. 2A and Fig. S10). As RAFT agent concentration increases, the influence of kinetic control on free-volume reduction is expected to diminish once a limiting packing state is reached, resulting in a plateau in mechanical properties. Differences in free-volume distribution may have also contributed to the refractive-index variations observed between FRP and RAFT-printed materials in shadowgraph images, as refractive index is sensitive to density fluctuations and local heterogeneity within polymer networks. As shown in Fig. 2D and Fig. 3, prints produced with RAFT-containing resins appear less optically visible in shadowgraph, indicating a smaller refractive-index (RI) change[4]. As stated in the main manuscript (lines 268 - 269), this smaller RI change during printing likely contributed to the smoother surface finish observed in RAFT-based prints. This ability to influence free volume through polymerization kinetics therefore suggests a potential route for tuning optical properties, which may be advantageous for large-scale printing applications in term of surface finishing and will be explored in future work.

Thus, the following text has been added to the main manuscript to expand our discussion as requested "Incorporation of RAFT agents retards the polymerization process, reducing kinetic trapping during network formation. This leads to a more efficiently packed polymer network with a lower and more homogeneous free-volume distribution and has been linked to enhanced mechanical performance in thermoset polymers[43]. Furthermore, as RAFT agent concentration increases, the influence of kinetic control on free-volume reduction is expected to diminish once a limiting packing state is reached, resulting in a plateau in mechanical properties observed in the data[44,45]" (lines 212-218).

References:

1. Childers, C. H., Hassan, M. K., Mauritz, K. A. & Wiggins, J. S. Molecular scale cure rate dependence of thermoset matrix polymers. Arab. J. Chem. 9, 206–218 (2016).

2. Zhang, H. J. et al. Effect of free-volume holes on mechanical properties of epoxy resins studied by positron annihilation and PVT experiments. Polymer 98, 47–55 (2016).

3. Odegard, G. M. & Bandyopadhyay, A. Physical aging of epoxy polymers and their composites. J. Polym. Sci. B: Polym. Phys. 49, 1695–1716 (2011).

4. Chung Li, C., Toombs, J. & Taylor, H. Tomographic color Schlieren refractive index mapping for computed axial lithography. in Symposium on Computational Fabrication 1–7 (ACM, Virtual Event USA, 2020). doi:10.1145/3424630.3425421.

3) Why different colors were observed in different printed items in Figure 4 and Figure 5?

The *observed coloration arises from three primary factors:*

(a) *In Fig. 4, the printed structures contain internal channels into which a colored liquid (green or blue food dye) was intentionally introduced via syringe. This dye was used solely to visually confirm the successful fabrication and continuity of the hollow features and does not reflect the intrinsic color of the printed material.*

(b) *In Fig. 4D and Fig. 5, the observed faint "pink" coloration originates from the incorporation of the RAFT agent into the PETRA-based resin formulation. The RAFT agent used (CPBD) comes in the form of red liquid or solid. Thus, its addition often imparts a slight pink coloration to the polymers formed using this control agent. This effect is more pronounced in the structures shown in Fig. 5 because they are bulky, solid objects without hollow features; consequently, the intrinsic coloration of the RAFT-containing polymer is more readily visible.*

(c) When RAFT agents are combined with PETRA-based formulations, a faint pink coloration is observed in the printed objects, whereas this effect is not apparent for some other monomer systems, for example the Polyglycerol 4 acrylate and PEGDA 700 as shown in Fig. 4C and E. This difference was proposed to reflect either (a) a reduced RAFT concentration required to control the polymerization of that specific monomer and/or (b) variations in the chemical environment of the thiocarbonylthio group, including differences in monomer and RAFT interactions, which can influence the optical absorption and perceived color of the final polymer.

4) Figure 3: why the position of different size balls is different in CRP and RAFT printing?

As *per our response to Reviewer 2 Comment 1, the elevated temperatures in the non-RAFT vial result in thermal currents emerging within the mixture. As a result, the objects fabricated with free radical polymerization rise on these convection currents and move upwards (discussed in main manuscript line 258 to 267). When using RAFT, these currents are*

minimised and the objects remain in their intended location. Consequently, we see different object positions when using FRP or RAFT.

5) The resolution data of RAFT CAL 3D printing should be provided in the manuscript.

One of the aims of this work was to increase volumetric packing density and enable multipart printing. Accordingly, the relevant measure of resolution was defined as the minimum distance between independently resolved parts printed simultaneously, which was determined to be 150 µm using the RAFT system, corresponding to three projected pixels (voxels) as seen in figure 3. This value should be interpreted, within the context of the optical arrangement used, as the achievable resolution. This resolution distance is inherently limited by the projector pixel size and its ability to deliver spatially precise light doses. A projection system with smaller pixels and finer intensity control would be expected to improve spatial fidelity and, consequently, the minimum resolvable separation. Thus this text was added to the manuscript to highlight this resolution hypothesis. "An aim of this work was to increase volumetric packing density and enable multipart printing. Thus, the relevant measure of resolution was defined as the minimum distance that could be achieved between parts printed simultaneously that remained independently resolved. For the apparatus used in this study, this was determined to be 150 µm using the RAFT system, corresponding to three projected pixels (voxels), as seen in Fig. 3." in lines 272-276

While these results demonstrate that high resolution can be achieved for simultaneous multipart printing, minimum feature size was not systematically investigated, as this was not the primary objective of the manuscript. Nevertheless, given the increased kinetic control afforded by RAFT-mediated polymerization, it is plausible that this approach could enable improved feature resolution relative to conventional free-radical systems[1]. Verification of this hypothesis will require dedicated studies using higher-resolution projection optics and targeted feature-size analysis.

References

1. *Green, B. J. & Guymon, C. A. Modification of mechanical properties and resolution of printed stereolithographic objects through RAFT agent incorporation. Addit. Manuf. 27, 20–31 (2019).*
